# Holocene centennial to millennial shifts in North-Atlantic storminess and ocean dynamics

**DOI:** 10.1038/s41598-018-29949-8

**Published:** 2018-08-24

**Authors:** Jérôme Goslin, Mikkel Fruergaard, Lasse Sander, Mariusz Gałka, Laurie Menviel, Johannes Monkenbusch, Nicolas Thibault, Lars B. Clemmensen

**Affiliations:** 1 Department of Geosciences and Natural Resource Management, University of Copenhagen, Copenhagen, Denmark; 2 Alfred-Wegener-Institute, Wadden Sea Research Station, List/Sylt, Germany; 3 Department of Biogeography and Palaeoecology, Adam Mickiewicz University, Poznan, Poland; 4 Climate Change Research Centre, University of New South Wales, Sydney, Australia

**Keywords:** Palaeoclimate, Natural hazards

## Abstract

The forcing mechanisms responsible for centennial to millennial variability of mid-latitude storminess are still poorly understood. On decadal scales, the present-day geographic variability of North-Atlantic storminess responds to latitudinal shifts of the North-Atlantic westerly wind-belt under the prime control of the North-Atlantic Oscillation (NAO). An equivalent mechanism operating at centennial to millennial time scales during the Holocene is still to be ascertained, especially owing to the lack of high-resolution and continuous records of past-storminess extending far enough in time. Here we present a reconstruction of past storminess activity based on a high-resolution record of wind-blown sand retrieved from a near-coastal wetland. Our record extends back to ca. 10,000 B.P. and allows to continuously document fluctuations in the frequency of Holocene storm-force winds at our study-site at a mean high temporal resolution of 40 years. Large similarities between our record and palaeo-oceanographic records of Holocene climate changes in the North-Atlantic suggest that our past-storminess record reproduces a signal of significance for the North-Eastern Atlantic realm. We find that Holocene North-Atlantic storminess is dominated by robust millennial (≈2,200-year) to centennial (≈450, 300 and 200-year) periodicities. These changes in storminess were accompanied by changes in the precipitation regimes over northern Europe, evidencing large-scale shifts in the latitudinal positions of the Atlantic westerlies akin to present-day NAO patterns. We propose that these shifts originate from changes in the position and extent of the Azores high-pressure system and Polar vortex, as supported by climate model simulations. Finally, we demonstrate that enhanced zonal storminess activity over the North-Atlantic was the driver of millennial and centennial-scale changes in North-Atlantic oceanic circulation, while ocean dynamics most likely influenced back the atmospheric circulation at millennial time-scales. This may vouch for the instrumental role played by North-Atlantic storminess in triggering abrupt climate change at centennial scales during the Holocene.

## Introduction

Westerly wind-belts and associated storminess are prime agents of North-Atlantic mid-latitude climate modulation. During recent years, efforts have been made to predict changes in storminess activity related to present and future climate-change notably through the use of coupled ocean-atmosphere models. However, there is currently no clear consensus on the future latitudinal position of extra-tropical storm tracks. Indeed, climate projections alternatively suggest that a northward or a southward shift of extra-tropical storm-tracks over the North-Atlantic may be experienced^1^, bearing large consequences for the builing of reliable strategies of adaptation to future climate change in North-western Europe.

At present, a large proportion of the climate variability in North-western Europe is controlled by the North Atlantic Oscillation (NAO)^2^. The NAO especially influences the latitudinal position of the westerly wind-belt and associated storminess over the North-Atlantic. A common paradigm is that positive phases of the NAO (NAO+) induce a zonal northerly located Atlantic Westerly Jet that drives storm-tracks and humid air masses over northern Europe, while the westerly circulation over southern Europe is blocked^3^. On short (monthly to decadal) time-scales, tight connections have been evidenced between NAO variability, activity of westerly winds and oceanic circulation in the North-Atlantic. NAO+ situations were for instance shown responsible for meridian displacements of the subpolar (SPG) and subtropical (STG) gyres in connection with the onset of a northward relocated and strengthened westerly wind stress (e.g.^4^). Modelling studies^5^ suggested that prolonged NAO+ situations and associated wind anomalies over the North Atlantic could have been instrumental in triggering of the Holocene abrupt cold climatic anomalies through oceanic feedbacks^6^, thus promoting storminess to a central role in the modulation of the Earth’s climate. However, the control exerted by the NAO on the position of North-Atlantic westerly storm tracks as well as the mechanisms controlling the NAO variability have been repeatedly discussed^7^ and are currently challenged. An emerging view is that the leading role of the NAO may have been overestimated, and that other modes of atmospheric variability such as the East-Atlantic pattern (EA), responsible for atmospheric blocking events over the North-eastern Atlantic, and the Scandinavian (SCAND) pattern may play important roles in controlling climate variability and storminess over North-Western Europe^8–10^. A three-poles linking blocking events (as part of the EA pattern), Total Solar Irradiance and Subpolar Gyre (SPG) dynamics has notably been suggested to have dominated atmospheric and climate variability at decadal to centennial scale over the last millennium^8^.

Better understanding the links between NAO variability and storminess over long time-scales therefore remains a central question, as it would allow to refine the projections produced by climate-models and to better address the challenges raised by ongoing climate-change. Up to now, the simulations of climate-models have been mostly validated against records of recent and historical meteorological data ranging from years to a few centuries in length^11^. Validation by robust storminess data extending over pluri-millennial time-periods is still lacking to ascertain the long-term spatio-temporal variability of storminess predicted by the models. How and under which control North-Atlantic storminess has varied in the past at shorter centennial-scales is still poorly understood. A reason for this may be that traditional wave-induced palaeo storminess indicators can hardly produce storm reconstructions of sufficiently wide spatial significance and of sufficiently high temporal resolution over the Holocene^12^. During the last decade however, promising reconstructions of past storminess have been derived from proxies of past aeolian activity (aeolian sand influx in near-coastal and perched peat-bogs), notably in Northern Europe and Scandinavia^13–15^ but also worldwide^16^. The use of aeolian sand influxes preserved within low-energy organic sedimentary sinks offers numerous advantages over the proxies of storminess derived from wave-influenced coastal stratigraphies. On the one hand, peatlands are very much stable sedimentary environments and offer the opportunity (i) to derive continuous records that can be precisely dated and (ii) to obtain perfectly synchronous palaeo-environmental data on the same core^17^. On the other hand, the process of aeolian sand transport is controlled to a high degree by the relationships between wind-energy and the size of the material available for transport, so that it has become possible to evaluate the occurrence of winds of different intensities over time^13^. Despite promising results, the use of aeolian proxies of past-storminess activity has for now remained rather limited.

**Figure 1:**
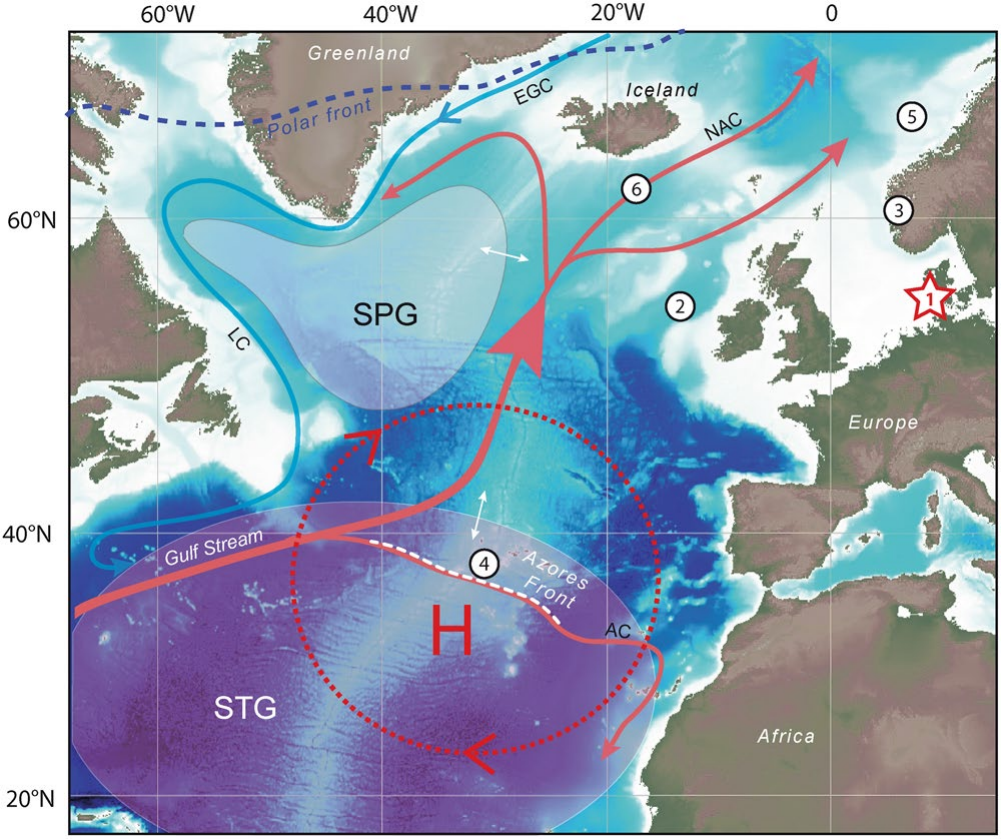
Location of the palaeo-climatic records used in this study and of the main features of atmospheric and oceanic circulation discussed in the manuscript. (1) Location of our study site, Filsø, western Denmark. (2) to (6) Location of the palaeo-climatic and palaeo-oceanographic records which are compared to the storminess record produced in the present study. (2) Record of stacked Ice-Rafted Debris (IRD^31^), (3) Record of precipitation change in SW Norway^54^, (4) Record of the position of the Azores front (AF^53^), (5) Record of the activity of the North Atlantic Current (NAC^57^), (6) Record of the extent and strength of the Sub-Polar Gyre (SPG^6^). The main oceanic currents of the North Atlantic are indicated (North Atlantic Current: NAC, Azores Current: AC, East Greenland Current: EGC, Azores current: AC). The letter H (L) and the dotted red (blue) line show the schematized positions and extents of the Azores (Polar vortex) High (Low) pressure systems, respectively. The mean location of the oceanic Sub-Polar Gyre (SPG) and Sub-Tropical Gyre (STG) are also depicted. White arrows refer to the changes in extent and position of the SPG and STG as discussed in the manuscript. Figure was designed using Adobe Illustrator CS3 (https://www.adobe.com) using open-access GEBCO bathymetric and topographic charts (General Bathymetric Chart of the Oceans, https://www.gebco.net).

Here, we investigate the links between storminess in North-western Europe and ocean-atmosphere variability in the North Atlantic during the Holocene on the basis of past influxes of aeolian sand recorded in the sedimentary sequence of Filsø, a coastal wetland of Western Denmark (Fig. 1, Supp. Info. 1). The record extends back to the early Holocene (ca. 10,200 B.P.), making it the longest continuous and high-resolution record of past storminess for North-western Europe. Combined with palaeo-oceanographic records available for the North Atlantic (Fig. 1), this unique record allows us to display and discuss the forcing mechanisms behind the variability of North-eastern Atlantic Holocene wind climate.

### Regional settings

The study area forms an ideal candidate for storminess reconstruction on the basis of wind-transported material. At an early stage of evolution, Filsø formed upon a glacio-fluvial topography and is considered to have experienced marine conditions in its deepest parts during the transgressive maximum of the regional mid-Holocene relative sea-level (around 7,000 B.P.^18–20^). From 7,000 B.P., the connection to the open North Sea closed rapidly by barrier-spit development driven by an intense southward-orientated longshore wave transport^19,21^ fed with the products of the erosion of local moraines, proglacial glacio-fluvial deposits and smoother periglacial landscapes^18,21^. A low-energy organic depositional environment settled fast behind this barrier^22^. At the same time, stacked beach ridge systems formed and prograded towards the west (seaward) and south under a continued and high sediment supply, completely isolating Filsø from direct marine influences^19,21^. Thick aeolian sand dunes subsequently developed upon the wave-built deposits and covered most of the area. First local occurrences of aeolian sand deposition were recorded south of our study site ca. 15,000 B.P., most probably originating from the reworking of glacio-fluvial sands of Saalian and Weichselian ages (as part of the European Sand Belt^22–24^). Periods of intense large-scale inland aeolian sand movement and coastal dune formation were then observed in western Denmark at around 8,000 B.P., 6,000 B.P. and ca. 4,300, 3,450, 2,800 and 2,500 B.P.^19,22,23,25,26^. At present, prevailing winds blow from the south-western to western sectors along the west coast of Denmark^27^. Storm activity is rather high, with winds of gale-force (8 on the Beaufort scale) and above occurring at annual frequencies of 5–10%. The drift potential reaches 1510 vector units (wind data in knots), thus making the wind-climate a climate of high-energy^28^.

### Stratigraphic record

The stratigraphy of the site was reconstructed based on two cores (F06 and F02) retrieved from the western part of Filsø (55°42′N, 8°13′E, Fig. 1 and Supp. Info. 1 and 2) which were linked using µ-XRF data (Supp. Info. 2). The chronology was built using ten AMS ^14^C dates and two optically-stimulated luminescence (OSL) ages (Supp. Info. 2 and 3).

**Figure 2:**
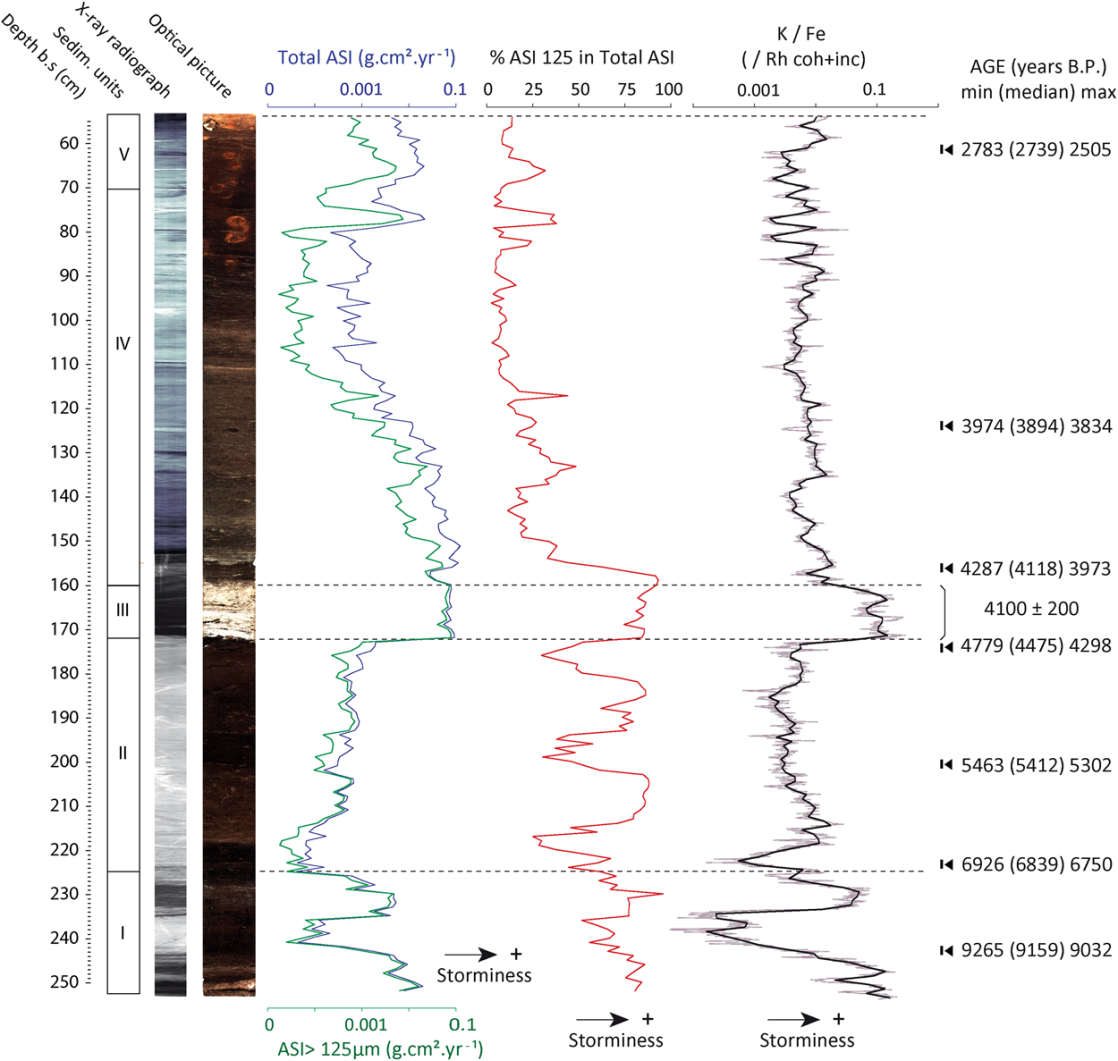
Stratigraphy, sand content and geochemical characteristics of core F06. Depth is in cm below surface. From left to right: Sedimentary units, X-ray radiograph of the core, optical picture of the core, Total (all sand >63 µm) and “coarse” (>125 µm) Aeolian Sand Influxes (ASI), percentage of sand >125 µm within the total ASI, downcore K/Fe ratio (black line is a 10 mm average centered on the depth used for ASI calculations), Ages in cal. years B.P.

The reconstruction of past-storminess shown in the present study was derived from core F06 only. The inferred age of the sediment in the core F06 spans from 10,200 B.P. to about 2,500 B.P. It was sampled with a mean resolution of 40 years (i.e. 1-cm sampling interval; Fig. 2, Supp. Info. 2). Core F06 can be subdivided into four main sedimentary units (Fig. 2): (i) Bottom units I and II (10,200-6,800 and 6,800-4,300 B.P., respectively) are composed of well-decomposed gyttja deposits progressively gaining organic content towards the top. Based on the paleo-geographical context of the site, the plant macrofossil assemblages for this unit (Supp. Info. 4) and with reference to other reconstructions from the area^22^, we believe that unit I deposited in a small freshwater pond which surely developed within a depression in the undulated antecedent low-gradient glacio-fluvial topography. This pond evolved into a back-barrier freshwater lacustrine environment (unit II), that the plant macrofossils assemblages show to have remained very shallow with water-depths most likely lower than 1 m^29^ (Supp. Info. 4). At a depth of 1.72 m below core top (−1.43 m DVR 90), there is a sharp transition to a 15 cm-thick bed of dune-sand (unit III, Fig. 2, Supp. Info. 2) which was dated to 4100 ± 200 B.P. and undoubtedly corresponds to the period of enhanced aeolian activity and intense dune movement identified for the same period along the entire western coast of Denmark^26^. Above this sand layer, a 1 m-thick layer of a well-humified *Sphagnum*-peat (unit IV, Fig. 2) is encountered which is primarily composed of *Sphagnum* *teres* and *Sphagnum* *palustre* (Supp. Info. 4). The topmost 0.55 m of the core were disturbed by agricultural practices as evidenced by a clear ploughing surface (Supp. Info. 2) and were not considered in the investigation (water level in Filsø was artificially lowered for land reclamation around the mid-1800s until 2012 when the site was turned back into a lake).

### Source of sand

There are several means by which sand-sized grains can be deposited within terrestrial organic sedimentary sinks. These include aeolian transport, remobilization of lake-shore material, fluvial input or mass wasting^17^. Our study site being concerned with two small rivers entering the site in its south-eastern termination, the source of the sand material found within core F06 organic sediments had to be tested for its aeolian origin so that the sand influx could be subsequently used as a trustful proxy of past aeolian activity and westerly storminess at the site. To do so, we used a combination of geochemical (µ-XRF) and paleo-ecological indicators (see the method section).

Modern coastal, dunes and river surface sands were sampled around the study site (Supp. Info. 5) and analysed for their composition in major and trace elements. The results revealed significant geochemical differences between coastal (beach and dunes) and river sand populations. The former are characterized by high Si and K contents, while the latter are marked by high Fe and Mn contents (Supp. Info. 5). Thus, the very good correlation observed between the ASI at our site and peaks in K/Fe ratio (Spearman correlation = 0.46, p < 0.0001 between K/Fe and Total ASI across unit I and II) suggests that the sand found in the core is primarily composed of coastal sand (Fig. 2). This statement is reinforced by the paleo-geographical information obtained from the plant macro-fossils assemblages which show that the site evolved into a very shallow lake (water-depth <1 m, 22, Supp. Info. 4) between ca. 7,000 and 4,300 B.P. It is thus unlikely that wave energy in the lake could have been high enough to provoke an erosion of the shores and large sand redistributions. As for the upper part of the sequence, the *Sphagnum* peat-bog character of the depositional environment (Supp. Info. 4) indicates that the occurrence of high-velocity water circulation can be ruled out at the coring site. The watershed of our study site is characterized by very gentle slopes (see elevations on Supp. Info. 1) and the two rivers of have very low transport competences. This makes it very unlikely that fluvial transport could have produced current velocities high enough to carry grain sizes >125 µm in suspension to the coring location. Based on these considerations, we infer that the sedimentary sequence of core F06 continuously recorded influx of aeolian sand blown downwind by storm-winds from NW to SW sectors between 10,200 and 2,500 B.P.

### Storminess record

The sedimentary record of core F06 shows repetitive fluctuations between periods of higher and lower input of aeolian sand to the site between 10,200 and 2,500 B.P. The bottom part of the sequence (unit I, ca. 10,200-6,800 B.P.), is first characterized by a marked succession of two wide peaks in both total and >125 µm ASI separated by a clear low. In this early stage of lake basin development, the sand may have mostly originated from the reworking of local glacio-fluvial sand sources (observed at the base of core F02, see Supp. Info. 2). From ca. 6,800 B.P. and onwards (unit II), the content of both total and >125 µm ASI progressively and steadily increased until the occurrence of the massive 4,300 B.P. sand invasion (unit III). The well sorted and rounded sand-grain population shows that the source of aeolian sand may have become dominated by coastal dunes during this period, following the establishment of coastal barriers directly west of the site after the mid-Holocene deceleration of the relative sea-level rise^19,21^. The gradual increase of aeolian sand influx across this unit II is most likely the expression of the transgressive coastline and coastal dunes systems moving eastward (and upwards), thus getting closer to the coring-site. The most prominent features of unit II are the well-defined and cyclic alternations between periods of very high (above ca. 75%) and low (25%) proportion of sand >125 µm in the total ASI (Fig. 2). Following the deposition of the sandy unit III (4,300-4,100 B.P.) and throughout most of unit IV, we observe a stable decrease of ASI, and notably of the ASI >125 µm before it rises again sharply around 3,200 B.P. An abrupt drop in the proportion of coarse sand in the total ASI to the site occurs at the transition between unit III and unit IV, with mean percentages of sand >125 µm dropping at around 15-20% and peaks hardly reaching 50% of the total ASI. Clear and well-defined peaks in the percentage of sand >125 µm nonetheless appear, still showing very good consistency with the peaks in total sand influx and suggesting periods of intensified storminess activity. In opposition to the steady increase of ASI observed throughout unit II, which we link to the mid-Holocene transgression, we argue that the gradual decrease in ASI shown for unit IV may be the expression of an intense westward (seaward) progradation undergone by the exposed coastline after 4,300 cal. B.P.^19,21^.

The sedimentary record from Filsø can be divided into eight main periods of increased storminess (“Filsø Storm Periods” FSP) centred on 9,700-9,100 (FSP1), 8,800-8,600 (FSP2), 8,300-7,100 (FSP3), 6,900-6,700 (FSP4), 6,400-5,500 (FSP5), 5,100-4,700 (FSP6), 4,400-3,800 (FSP7) and 3,300-2,800 B.P. (FSP7) (Fig. 5A). These results are in general agreement with the Holocene Storm Periods previously proposed for NW Europe^30^, but show two additional periods of marked storminess for the early Holocene (FSP1 and FSP2, Fig. 5-I). Furthermore, the continuous high-resolution storm record produced in the present study allow us to go further by depicting high-frequency centennial-scale changes in storm activity in the north-Atlantic during the Holocene which were never described with such accuracy.

### A multi-scale variability of Holocene North-Atlantic storminess activity

**Figure 3:**
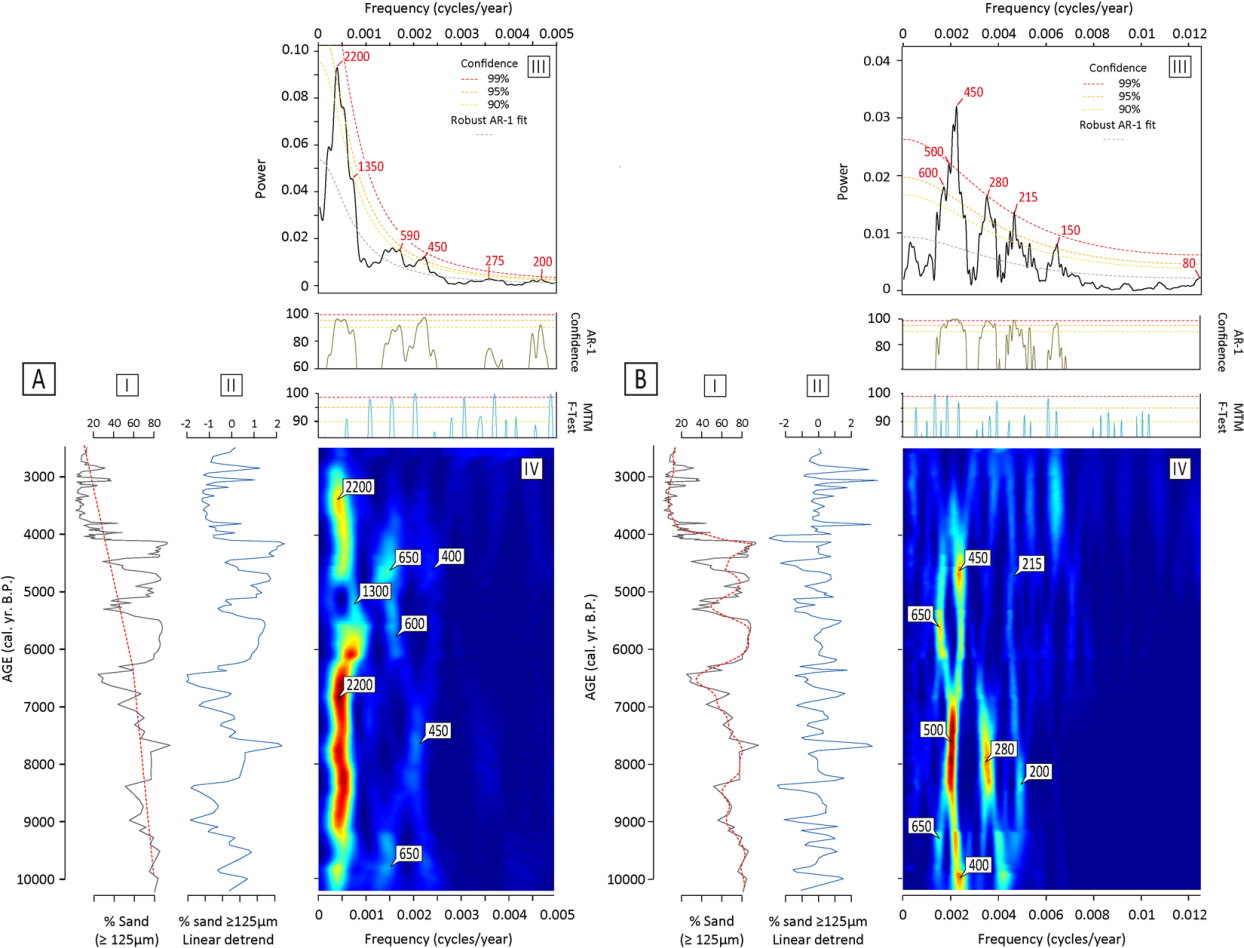
Spectral analyses^65^ and Evolutive Harmonic Analyses^66^ (EHA) analyses of the storminess record produced in this study. (**A**) Percentage of coarse sand in Total ASI vs. age (A-I, black line) detrended from its long-term trend (A-II, blue line), using the long-term trend (dotted red line on A-I, see methods). (**B**) Percentage of coarse sand vs. age (B-I, black line) detrended from its short-term trend (B-II, blue line), using the short-term trend (dotted red line on B-I, see methods). The power spectra of the detrended percentages of coarse-sand (A-II and B-II) are presented on the top right diagrams A-III and B-III. The red numbers on power spectra diagrams (A-III and B-III) mark the main periodicities present in our storminess record at the 99% confidence level. The evolutions of these latter periodicities with time are shown on the associated EHA diagrams (A-IV and B-IV). The blue to red color scale on A-IV and B-IV indicates the power of the frequencies (Blue-low power, Red-high power) and their evolution through time (in years B.P.). The white numbers on the EHA diagrams are reading guides that help to locate and follow the evolution with time of the dominant periodicities shown on the diagrams A-III and B-III (in years).

To evaluate the modulations of the north-eastern Atlantic storm activity over long- (millennial) and shorter-term (pluri-decadal to centennial) time periods, we performed time-series analyses on our records (Fig. 3 and methods). Spectral analyses were made to evidence the dominating wavelengths comprised in our signal (Fig. 3A-III,B-III), while Evolutive Harmonic Analyses (EHA) allowed us to follow changes in these periodicities through time (Fig. 3A-IV,B-IV). Spectral analysis of the lower frequencies shows storminess to be composed of five periodicities of ≈2,200, ≈1,350, ≈590, ≈450 and ≈200 years at the 90% significance level (Fig. 3A-III), the ≈2,200-yr period showing the strongest power. As higher frequencies are concerned, strong wavelengths of ≈600-, ≈500-, ≈450-, ≈280-, ≈200-, ≈150- and ≈80-yr periods are evidenced (Fig. 3B-III), demonstrating the persistence of pluri-decadal to centennial scale modulations of North Atlantic storminess during the Holocene. EHA show variable stationarity of these millennial and centennial periodicities. A relatively wide band of quasi-periodic variability containing the dominant ≈2,200-yr period clearly dominates the signal over most of the Holocene (Fig. 3A-IV). A notable non-stationarity of the spectral signature nonetheless appears between ≈6,000 and ≈4,500 B.P., when the signal observes a marked excursion toward shorter periods of ≈1,300 years (Fig. 3A-IV). This temporary excursion towards a ≈1,300-yr mode of periodicity at the mid-Holocene seems accompanied by a divergence in the high-frequencies wavelengths characterizing the storminess signal during the early-Holocene (Fig. 3B-IV). Indeed, from ca. 10,200 to ca. 6,800 B.P, EHA show our storm record to be dominated by robust quasi-stationary ≈400 to 500-yr and ≈300-yr wavelengths (Fig. 3B-IV). A clear shift operates around 6,800-6,500 B.P. when the ≈280-yr periodicity is observed to progressively vanish while the ≈500-yr period evolves towards a strong ≈650-yr and 450 ones.

Pluri-millennial periodicities have been repeatedly documented in Holocene palaeo-environmental studies of the North Atlantic domain. The origins of these latter have been intensively discussed with regards to external (solar) and internal (oceanic/atmospheric) variability^30–35^. Periodicities of ca. 2000-2500 years have been proposed to be strongly correlated with variations in the total solar irradiance^34,36^. The persistence of a clear and stationary ≈2,200-yr periodicity in our storm-record over most of the Holocene (Fig. 3A-IV) thus bring in contention that solar forcing may have been the main driver of the functioning of the climate system and storminess activity at a ca. 2,500-yr pace over the Holocene. Shorter periodicities of ≈1,300–1,500 yrs were suggested to be either sub-harmonics of the 2,500- and 1,000-yr cycles^32^ or the expression of a massive reorganization of North Atlantic thermohaline circulation at that time^37^. The non-stationarity of the low-frequencies we observe within our storm record between ca. 6,000-4,500 B.P., characterized by an excursion of the signal towards a shorter period of ≈1,300 yrs may thus be the expression of a temporary overtake of the influence of solar forcing on climate by the mid-Holocene stabilization ocean-atmosphere internal modes of variability as suggested by previous studies^30,37–40^.

Interpreting the origin of the pluri-centennial periodicities evidenced by spectral analyses in our storminess record is more difficult, owing to consequent trade-offs between the several internal forcing factors susceptible to have been active at these time-scales^41–43^. Periodicities of around ≈500, ≈400, ≈300 and ≈200 yrs have all been suggested to reflect fluctuations in the total solar irradiance^42–46^. The EHA especially depicts a great stationarity of the ≈200-yr period in our storm record throughout most of the Holocene, suggesting that it could have been the main mode of variability of North-Atlantic storminess at centennial scale. The ≈200-yr period is traditionally attributed to be the expression of the Suess-DeVries solar cycle (208-yr period^44,47^). How variations in solar irradiance are transmitted to atmospheric and surface wind circulation remain for now rather unclear. Solar irradiance may be transmitted to the westerly jet activity throughout a “top-down” coupling between the stratosphere and the troposphere^48^. Models of the effect of solar ultraviolet irradiance on North-hemisphere winds showed that this may be instrumental in fostering baroclinic gradients responsible for NAO variability at decadal scales, and demonstrated that solar minima may translate into strengthened surface mid-latitude westerlies activity during the winter season^48–50^. The amplification of solar variability through ocean-feedback patterns have also been suggested to possibly occur by changes in the thermohaline circulation^51^ and by solar heating of the sea-surface being transmitted to the atmosphere by “bottom-up” sea-air coupling^52^. Interestingly, the loss of power of the ≈200-yr period between ca. 6,500 and 4,500 yrs B.P. (Fig. 3B) occurs simultaneously with large reorganizations of the oceanic circulation reconstructed from proxy-records for the mid-Holocene, such as the onset of warm subsurface water transport from the tropics to the subpolar regions^53^ and the weakening of subpolar gyre circulation^37^, suggesting that these mechanisms may be linked to each other either by control links or by being the product of a common driver.

### Zonality of Holocene North-Atlantic storminess controlled by atmospheric variability

Synchronous variations between our record of storminess and proxy-records of precipitations available from Scandinavia and southern Europe confirm that variations in storminess activity may have originated from persistent north to south fluctuations in the latitudinal position of North Atlantic westerly winds and storm-tracks during the Holocene (“seesaw” patterns). Periods of increased storminess documented by our record show for instance good agreement with the periods of increased humidity identified in south-western Norway for the Holocene^54^, implying that both regions recorded concurrent large-scale changes in the delivery of humid air masses during the Holocene (Fig. 5A). On the contrary, the abrupt drop in storminess shown in our record around 6,500 B.P. was accompanied by a rapid onset of more humid conditions over Portuguese wetlands as well as by a shift from an extra-tropical to a tropical provenance of the humid air masses affecting the Iberian Peninsula^55^. Thus, we infer that the marked drop of storminess observed at this period was controlled by a southward relocation of the Northern Atlantic westerly wind belt. In reference to the mechanisms behind present-day NAO variability, it is reasonable to hypothesize that such changes in the position of Holocene North-Atlantic westerly winds may have been produced by large-scale variations in the temperatures and pressure gradients across the North Atlantic under the primary control of changes in the position and extent of the Azores High.

**Figure 4:**
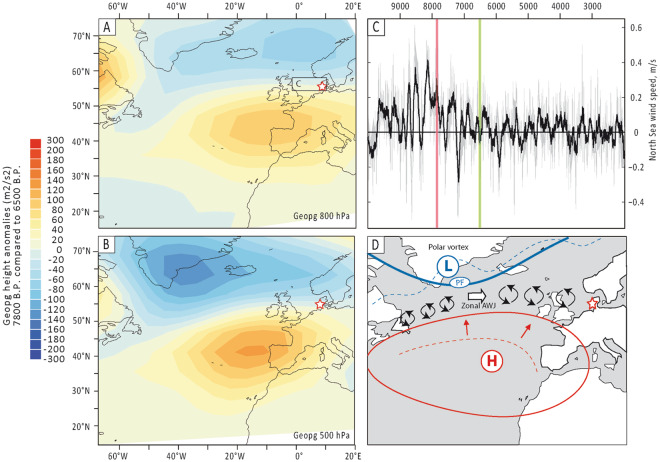
(**A** to **C**) Transient simulations of the Holocene performed with the Earth system model LOVECLIM^56^. (**A**) Differences in the geopotential heights at 800 hPa between the ca. 7,800 B.P. period (stormy period) and the ca. 6,500 B.P. period (non-stormy period) calculated by substracting the geopotential heights at 800 hPa at 6,500 yrs B.P. from those at  7,800 yrs B.P. Based on 100 years averages (time+/− 50 years). (**B**) Same than (**A**) but at  500 hPa. **(C)** 800 hPa Wind speed (m.s^−1^) anomalies averaged over 0°-12°E and 54°N-58°N (see boxes on A and B). Red and green vertical bars respectively locate the periods of High- and Low-storminess used for (**A**) and (**B**). The mean over the last 12,000 years is taken as reference. **(D)** Conceptual drawing of the configuration of atmospheric pressure centre on the North-Atlantic during periods of enhanced storminess activity. The red and blues lines display hypothetical configurations of the extent of the Azores High and the Polar Vortex during periods of increased (plain lines) and decreased (dotted lines) storminess in the North-Atlantic. Vortex cells schematize cyclonic perturbations. Abbreviations: “H” stands for High pressure system (Azores High), “L” stands for Low pressure system (Polar vortex), “AWJ” indicates the Atlantic Westerly Jet, “PF” the Polar Front.

To test this hypothesis, we compared our reconstruction with a transient simulation of the Holocene performed with the Earth system model LOVECLIM^56^. The simulated 800 hPa wind speed averaged over the North Sea (Fig. 4C and Supp. Info. 6) and over the North-Atlantic (Supp. Info. 6) are in overall agreement with our data. Periods of increased storminess over the North Atlantic also correspond to periods of relative increase of simulated precipitation over Northern Europe (Supp. Info. 6). In addition, the periods of enhanced storminess identified in our proxy record are associated with marked differences between the 800 hPa and 500 hPa geopotential heights around Iceland and the Azores (Supp. Info. 6), pointing towards periods of more positive NAO situations. In comparison to periods of low storminess activity, the periods of increased storminess are predicted by LOVECLIM model simulations to be characterized by prominent positive geopotential anomalies centred north of the Azores and extending over most of the North Atlantic and negative anomalies over southern Greenland (Fig. 4A,B). In other terms, LOVECLIM simulations support our hypothesis that Holocene periods of high storminess activity over Northern Europe were associated with a stronger and more northward-located Azores high-pressure centre and with a strengthened polar vortex, in a configuration resembling present-day positive NAO-situations (Fig. 4D). Between these two strong centres of action, a narrow corridor most likely funnelled the Atlantic Westerly Jet and associated cyclonic perturbations, thus driving storm-tracks towards northern Europe (Fig. 4D).

### North-Atlantic storminess and ocean dynamics linkages

**Figure 5:**
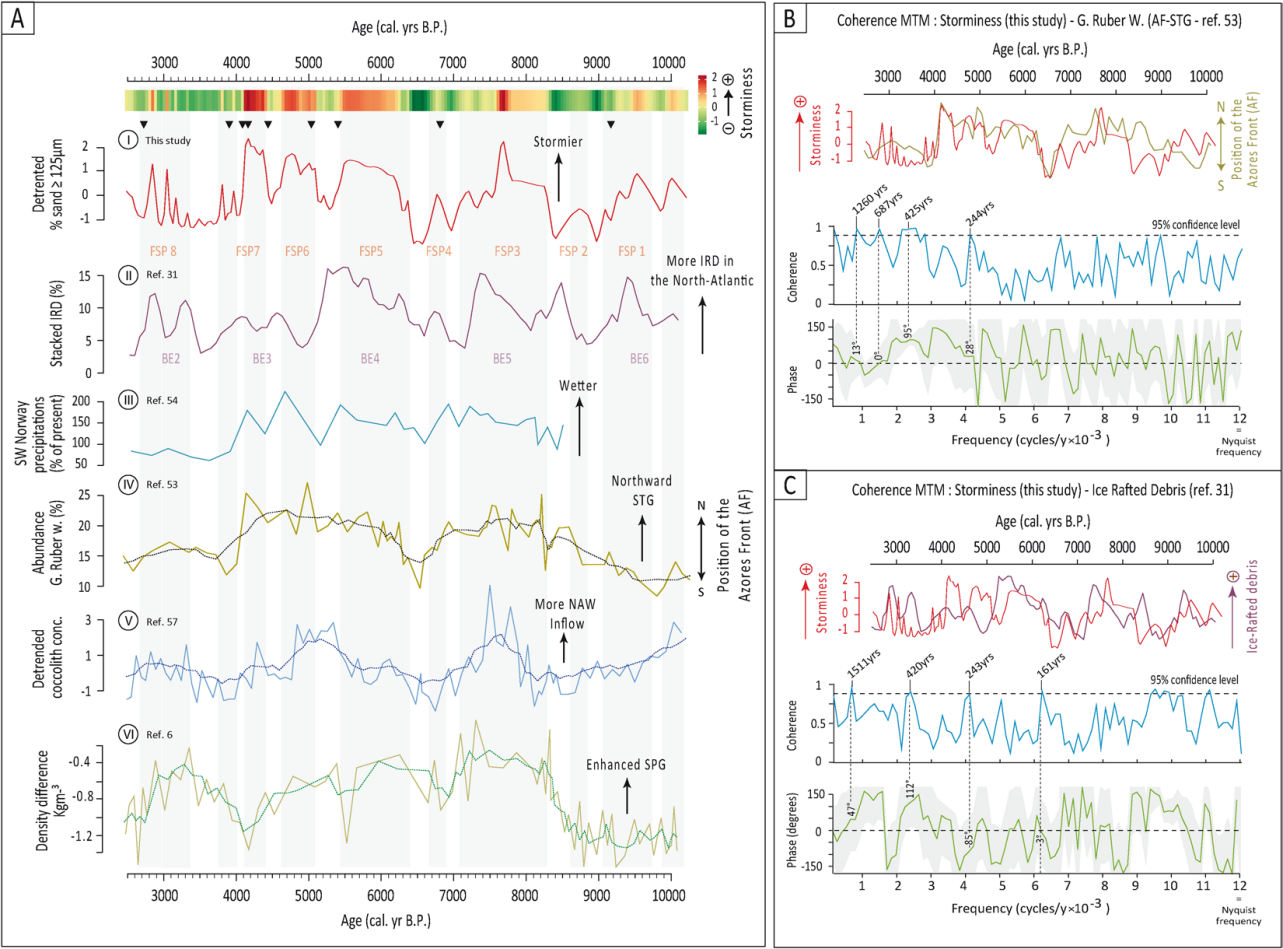
(**A**) (I) Storminess record from this study; (II) Hematite-stained grains record of Ice-Rafted Debris^31^; (III) Record of precipitation variability in SW Norway^54^; (IV) Record of the position of the Azores front^53^; (V) Record of the extent and strength of the Sub-Polar Gyre^6^ and (VI) Record of the activity of the North Atlantic Current^57^. The location of the proxy-records (I) to (VI) is indicated on Fig. 1 following the same numbering. (**B**) Multi-taper Coherence analysis of the storminess record produced in the present study and the position of the Azores front^53^. (**C**) Multi-taper Coherence analysis of the storminess record from this study and the percentages of stacked Ice-Rafted Debris^31^. On 5B and 5C, top diagrams show an overlay of the detrended times series analyzed by the CMTM^68^. Blue curves show the coherence (from 0 to 1) between the compared time-series in the frequency domain. The frequencies showing significant coherence are marked by their associated periods (in years). The phase-lag between the analyzed time series is shown by the green line, along with the phasing (in degrees) associated to each significant frequency/period. A phasing close to null shows the two analyzed time-series to be in phase at the considered frequency, a positive phasing shows a leading of the storminess record over the time-series to which it is compared.

Comparisons of our storminess record and palaeo-oceanographic records of the North Atlantic demonstrate the close links that existed between North-Atlantic variability and storminess activity at multi-temporal scales during the Holocene. A striking similarity stands out between our record and changes in the abundance of the planktonic foraminifera *Globigerinoides ruber* (white) *(G.ruber* w.) in a deep-marine core taken south of the Azores^53^ (Fig. 5A-IV,B). *G.ruber* w. being most abundant in the North-Atlantic Sub Tropical Gyre (STG), its abundance in marine shelf sediments is considered as a proxy for the extent of the STG and for the position of the Azores front (AF), high and low abundances revealing northward and southward position of the STG, respectively^53^. Periods of increased and decreased storminess evidenced by our storm record are concurrent with respective northward and southward shifts of the location of AF and thus of the STG throughout the Holocene on both long (millennial) and shorter (centennial) time-scales (Fig. 5A-I and A-IV). Multi-taper coherence analyses (CMTM, see methods) performed on our storm record and on the record of the abundance of G.ruber w. show the two series to share common periodicities of ≈ 1,300, ≈ 700, ≈ 400, ≈ 240 yrs, the highest coherence estimates being obtained for the ≈ 1,300, 700 and ≈ 400-yr periods (Fig. 5B). These results echo the same periodicities identified within our storminess record (Fig. 3), thus showing that both storminess and the position of the AF/STG most likely responded synchronously to one or several drivers varying at these periods. Which of atmospheric or oceanic variability was the first driver remains a challenging question to which our data cannot provide conclusive answers. The results of the coherence analyses between storminess and the proxy record of the extent of the STG^53^ nonetheless show clear positive phasings (of 13, 95 and 28-degrees, respectively) at both millennial and centennial time-scales (Fig. 5B). Such phasing suggests that changes in storminess led STG variability, in line with what is observed nowadays at inter-annual to decadal scales^4^. Smaller phases are obtained for ≈1,300 and 700-yr periods (13 and 0 degrees, respectively, Fig. 5B) being in favour of a bottom-up forcing of oceanic variability on atmospheric processes at millennial time-scales. A strong pacing at millennial to centennial time-scales is also evident for most of the Holocene between our storminess record and the activity of the sub-polar gyre (SPG) as expressed by differences in the density of the water masses^6^ (Fig. 5A-VI). During periods of increased storminess, density difference increases, indicating a stronger and more east-west elongated SPG, thus contributing more to the inflow of water to the north-eastern Atlantic through the NAC (Fig. 1). This is mirrored in the fair synchronism that appears at the ca. 2,500-year pace between our data and the strength of the North Atlantic Water inflow into the Norwegian Sea (Fig. 5A-V). CMTM analyses further show these two records to be coherent and in phase at the ≈300-yr period. We propose that strengthened storminess over the North Atlantic would have enhanced the activity of the North-Atlantic Current (NAC, Fig. 1) and brought warmer water to the Nordic seas^57^. This would have fostered large SST differences between the eastern and western part of this area, in line with the hypothesis that SST differences in the Nordic Seas relates to NAO variability^58^ and, maybe more importantly, to North-eastern Atlantic storminess activity. All in all, these results support the proposition recently made that persistent positive NAO-like situations and associated enhanced North Atlantic storminess during the Holocene may have been instrumental in amplifying or even triggering abrupt pluri-centennial cold events by strengthening the SPG and causing a freshening of the North Atlantic inflow and, in turn, the slowdown of the Atlantic Meridional Overturning Circulation^5,6^.

A nearly perfect peak to peak synchronism is also apparent between our record and the stacked record of Ice-Rafted Debris (IRD) reconstructed in the North Atlantic over the 10,200-2,500 B.P. period^31^ (“*Bond events*” 6, 5, 4 and 3, Noted “BE” on Fig. 5A-II). The supply of IRD to the North Atlantic was proposed to be due to an extensive cooling at polar and sup-polar latitudes of the Northern Hemisphere that could have strengthened the polar vortex and triggered the drifting of sea-ice, as a result of strong northerly winds^31^. An alternative explanation associates peaks of IRD in the North Atlantic to a dual strengthening of the East Greenland Current (EGC, Fig. 1) and of the North Atlantic Current (NAC, Fig. 1), under the joint influence of strong northerly winds caused by the presence of a strong Icelandic low-pressure system and of strengthened westerlies^59^. Multi-taper coherence analyses of our storm record and the input of IRD to the North-eastern Atlantic show the two series to be highly coherent at the 1,500-yr period with a phase lag of 47 degrees (Fig. 5C). Despite the uncertainties in the phasing produced by the CMTM analysis, this positive phasing suggests a leading of storminess over the IRD supply to the North-Atlantic, which is incidentally evident when looking at the two time-series superimposed on each other (Fig. 5C). Our results are therefore in favour of the IRD-events in the North Atlantic to be (at least partially) the result of strengthened and northward-located westerlies and storm-tracks in the North Atlantic during NAO-like situations that would have enhanced the SPG and helped spreading Ice-Rafted Debris over the North- western Atlantic.

## Conclusions

Our high-resolution record of aeolian sand influx to a coastal wetland in western Denmark allows the identification of multi-scale periodicities in North-Atlantic storminess over a large part of the Holocene. As such, it brings a significant contribution to the understanding of climate variability in the North-Atlantic realm during the past ten millennia. Our results further corroborate the hypothesis that North Atlantic westerlies and storm-tracks underwent millennial to centennial shifts in their latitudinal position during the Holocene. These changes appear to have been modulated by persistent long- and short-term NAO-like patterns, probably induced by changes in the position and strength of the Azores High pressure system and of the polar vortex. Furthermore, our findings stress that the close synergy observed today between North Atlantic oceanic circulation and the position of the westerlies wind belt in the North Atlantic also operated at millennial to centennial timescales during most of the Holocene. Storminess most likely drove the activity of North-Atlantic oceanic gyres at both millennial and centennial time-scales. This nexus highlights the major role of westerly wind belts not only as the products, but also as forcing-agents of climate variability. Further work would nonetheless be needed to disentangle the respective roles played by internal and external forcing agents in controlling the changes in the configuration of pressure centres and associated wind-shifts over the North-Atlantic.

## Methods

### Core collection and preparation

Two cores (F02, F06) were taken from a raft in the western part of Filsø (Western Denmark, see map in Supp. Info. 1) in September 2016 using a vibracoring system and aluminium tubes with an outer diameter of 80 mm. Cores were sealed and kept in a cold room at 4 °C until opening. Cores were then cut lengthwise and split in halves under subdued red-light conditions. Sand layers were sampled for OSL dating. The lithology/stratigraphy of the cores was described and pictures were taken.

### Dates and age-model

Two OSL dates were established on sand layers present at the bottom and in the middle of core F02. Ten radiocarbon dates were established on plant macrofossils on both F02 and F06 cores (Supp. Info. 3). All dates presented in the manuscript were calibrated using Intcal13 calibration program^60^ and are reported within a two-sigma confidence interval. Age-models were constructed using Clam age-modelling package in R software^61^. The age-model used in this study is a compound age-model constructed using ages from both cores F06 and F02 (Supp. Info. 2 and 3). Correlations between the two-cores were drawn on the basis of concurrent stratigraphic markers and peak-to-peak correspondence in K content (Supp. Info. 2). Sedimentation rates were calculated as the difference between two ages at a 1 cm-scale and consequently used for the calculation of the Aeolian Sand Influx index (see below).

### Storminess reconstruction

Storm activity was derived from the quantity of aeolian sand deposited within the organic sediment of the wetland. The first half of the core was cut into exact 1-cm thick slices. The dimensions of each slice were measured to obtain volumetric information. Slices were weighted, dried at 105 °C, cooled in a dessicator and re-weighted to obtain water content. Slices were then burnt at 550°, cooled in a dessicator and re-weighted to obtain loss-on-ignition (LOI). Post-LOI remains were washed with 10% hydrochloric acid and wet-sieved with 125 µm and 63 µm meshes to extract minerogenic content. The ≥ 63–< 125 µm (“fine” sand) and the ≥125 µm “coarse” sand fractions were then weighted. Building up on the Aeolian Sand Influx (ASI) method^13^, here we calculated the ASI by dividing the total sand weight comprised in each core slice by the sedimentation rate and the volume of the slice to obtain a sand influx to the site in g.cm^−3^.yr^−1^. Total ASI was calculated for all sand grains >63 µm, while ASI 125 was derived from the influx of sand grain of size >125 µm only. Following available laws of aeolian sand transport^62^, the influx of sand of grain-size ≥125 µm was considered to sign for storm events of wind speeds ≥22.5 m.s^−1^ at 10-m high (10 on the Beaufort scale and above). An additional indicator was used that is the percentage of sand of grain-size ≥125 µm comprised in the Total ASI, which was considered to sign for the frequency of storm events of wind speeds ≥22.5 m.s^−1^ at 10-m high (10 on the Beaufort scale and above). A storm index was then derived from the detrended the percentage of sand of grain-size ≥125 µm.

### Plant macro-remains

The paleo-ecological evolution of our site was investigated using plants macro-remains. 1-cm sediment slices were dispersed and sieved at 200 µm. Sieving residuals were investigated wet under the microscope and macro-remains were picked and identified. For each species, macro-remains are reported in term of occurrence at a 1-cm resolution (Supp. Info. 4).

### Tracing the sand sources

Core-F06 and F02 as well as modern beach, dune and watershed river domains surrounding our study site were analysed for their geochemical characteristics (Supp. Info. 5) using an ITRAX^TM^ core-scanner (Natural History Museum of Copenhagen) mounted with an Rh tube. As for downcore µ-XRF profiles, these were obtained at 1-mm increments with a current of 30 kV/50 mA and an exposure time of 35 seconds. Modern surface sand samples were analysed as discrete samples within round cups using the same settings. For both core and modern samples, raw XRF results in counts per second were divided by the sum of the Raleigh and Compton components (Rh coh + inc) to account for down-core variations in water and organic content^63^. The K/Fe ratio was chosen as the sand source indictor following the results given by the PCA investigation of the geochemical characteristics of modern surface sand samples (Supp. Info. 5). K was preferred over Si due to Si being potentially influenced by biogenic silica. Very good correlation between K and Ti ensured us that K was of minerogenic origin.

### Statistical analyses

Spectral analyses and wavelet transform analyses were performed on the coarse-sand fraction of the record using the Astrochron packages in R^64^. The signal was smoothed prior to spectral analysis by detrending the mean and the variance of the time-serie using a LOWESS smoothing. The variance was detrended by performing a Hilbert transform of the signal to get the instantaneous amplitude, calculating a LOWESS smoothing and by dividing the signal by the LOWESS smoothing. A Long-term linear detrending was performed using a LOWESS smoothing span of 1/1 and was applied to the record in order to investigate the low frequencies (Fig. 3). A LOWESS smoothing span of 1/15 was used on the record to investigate the high frequencies (Fig. 3). MTM spectrum analyses^65^ and Evolutive Harmonic Analyses (EHA^66^) were then ran on the interpolated series using a padding factor of 6 and a window size of 3,000 years. Confidence levels were estimated using a red noise fit (ML96^67^). Coherence Multi-Taper estimates analyses were performed between our data and literature palaeo-climatic/paleo-oceanographic time-series using cross-MTM method in Matlab (e.g.^68^). All time-series were linearly re-interpolated at 40 yrs from 2,488 to 10,200 yrs B.P. prior to the CMTM. The CMTM was estimated with 3 windows for the ASI-AF and ASI-NAC coherence estimations. Two windows were used for the ASI-Bond coherence estimate to better express the coherence at low frequencies (long time periods). All CMTM were run with 5000 Monte Carlo iterations for estimation of phase uncertainty.

## Electronic supplementary material

Supplementary information
