# Author Correction: Holocene centennial to millennial shifts in North-Atlantic storminess and ocean dynamics

**DOI:** 10.1038/s41598-020-69870-7

**Published:** 2020-08-06

**Authors:** Jérôme Goslin, Mikkel Fruergaard, Lasse Sander, Mariusz Gałka, Laurie Menviel, Johannes Monkenbusch, Nicolas Thibault, Lars B. Clemmensen

**Affiliations:** 1 Department of Geosciences and Natural Resource Management, University of Copenhagen, Copenhagen, Denmark; 2 Alfred-Wegener-Institute, Wadden Sea Research Station, List/Sylt, Germany; 3 Department of Biogeography and Palaeoecology, Adam Mickiewicz University, Poznan, Poland; 4 Climate Change Research Centre, University of New South Wales, Sydney, Australia

Correction to: *Scientific Reports*10.1038/s41598-018-29949-8, published online 24 August 2018

The original version of this Article contained errors.

The authors identified the following error in the published version of the Article: the topmost date of core F-02, serving as the uppermost constraint of the compound age-model was incorrectly given as 3,025–2,790 cal. yrs B.P and should be 2,783–2,505 cal. yrs B.P.

The authors calculated a new age-model using the correct date as the uppermost constraint. Aeolian Sand Influx (ASI) was recalculated using the corrected sedimentation rates and results were changed accordingly in all Figures. Spectral analysis and Evolutive Harmonic analyses were performed again based on the corrected time-series of ASI. Figure 3 was corrected accordingly. Coherence analyses between the authors’ and paleoceanography records were performed again. Figure 5B and 5C were corrected.

Minor adjustments have been made to the text to account for the errors in the original Article.

As a result of these corrections, in the Abstract,

“We find that Holocene North-Atlantic storminess is dominated by robust millennial (≈ 2,500-year) to centennial (≈ 400 and 200-year) periodicities.”

now reads:

“We find that Holocene North-Atlantic storminess is dominated by robust millennial (≈ 2,200-year) to centennial (≈ 450, 300 and 200-year) periodicities.”

Also in the Abstract,

“Finally, we demonstrate that enhanced zonal storminess activity over the North-Atlantic was the driver of centennial-scale changes in North-Atlantic oceanic circulation, while ocean dynamics most likely influenced back the atmospheric circulation at millennial time-scales.”

now reads:

“Finally, we demonstrate that enhanced zonal storminess activity over the North-Atlantic was the driver of millennial and centennial-scale changes in North-Atlantic oceanic circulation, while ocean dynamics most likely influenced back the atmospheric circulation at millennial time-scales.”

In the Introduction,

“The record extends back to the early Holocene (ca. 10,100 B.P.), making it the longest continuous and high-resolution record of past storminess for North-western Europe.”

now reads:

“The record extends back to the early Holocene (ca. 10,200 B.P.), making it the longest continuous and high-resolution record of past storminess for North-western Europe.”

In the Introduction section, under the subsection ‘Stratigraphic record’,

“The inferred age of the sediment in the core F06 spans from 10,100 B.P. to about 2,500 B.P. It was sampled with a mean resolution of 40 years (i.e. 1-cm sampling interval; Fig. 2, Supp. Info. 2). Core F06 can be subdivided into four main sedimentary units (Fig. 2): (i) Bottom units I and II (10,100–6,800 and 6,800–4,300 B.P., respectively) are composed of well-decomposed gyttja deposits progressively gaining organic content towards the top.”

now reads:

“The inferred age of the sediment in the core F06 spans from 10,200 B.P. to about 2,500 B.P. It was sampled with a mean resolution of 40 years (i.e. 1-cm sampling interval; Fig. 2, Supp. Info. 2). Core F06 can be subdivided into four main sedimentary units (Fig. 2): (i) Bottom units I and II (10,200–6,800 and 6,800–4,300 B.P., respectively) are composed of well-decomposed gyttja deposits progressively gaining organic content towards the top.”

The original version of Figure 2 contained errors and appears below as Figure 1. Figure 2 has been corrected in the original version of the Article where the curves of Total ASI and % ASI 125 have been replaced by curves of the same variables corrected from the age/depth model mistake. Topmost age in the rightmost column of the Figure has been replaced by the correct age.

**Figure 1:**
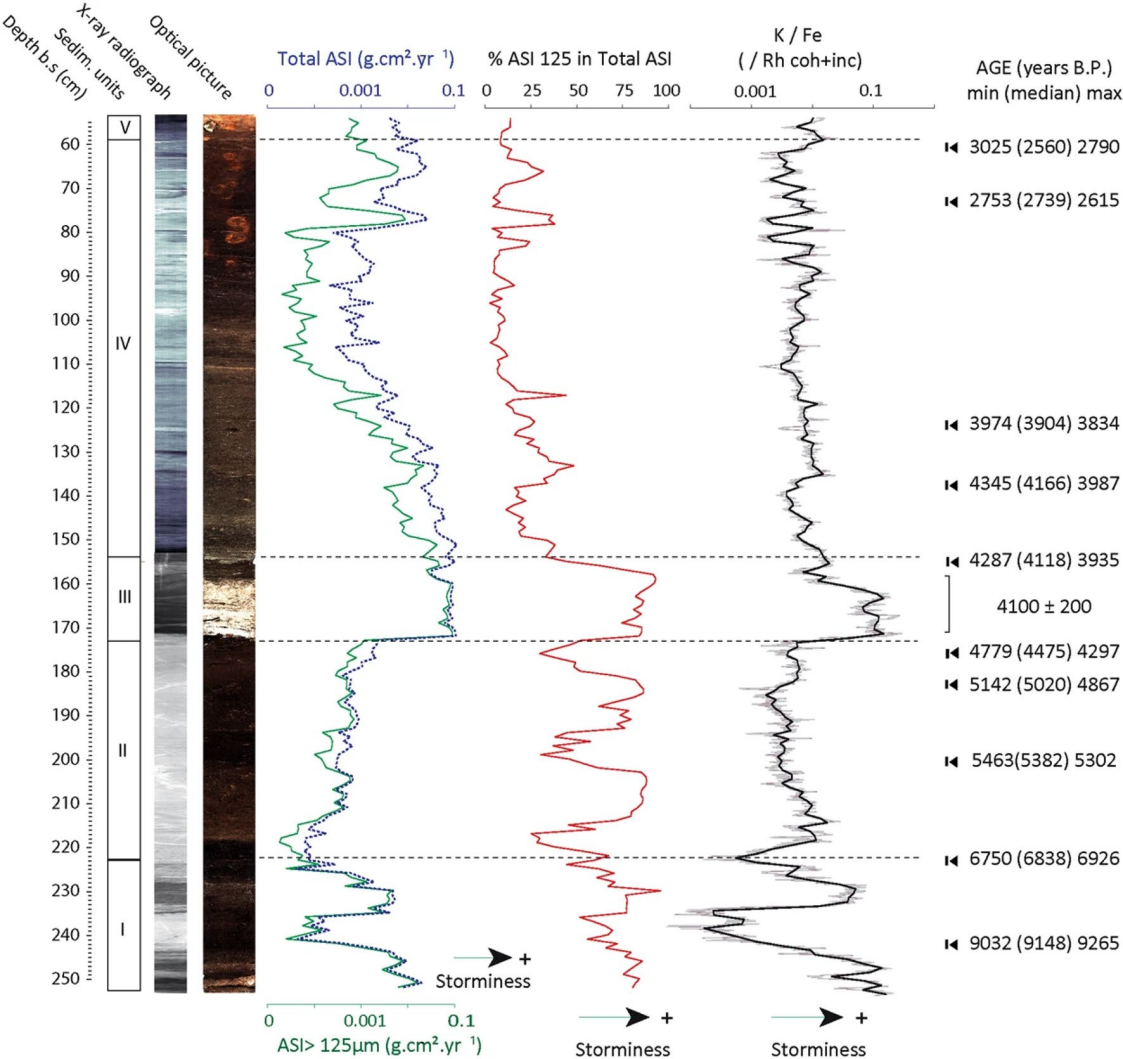
.

In the Introduction section, under the subsection ‘Source of sand’,

“Based on these considerations, we infer that the sedimentary sequence of core F06 continuously recorded influx of aeolian sand blown downwind by storm-winds from NW to SW sectors between 10,100 and 2,500 B.P.”

now reads:

“Based on these considerations, we infer that the sedimentary sequence of core F06 continuously recorded influx of aeolian sand blown downwind by storm-winds from NW to SW sectors between 10,200 and 2,500 B.P.”

In the Introduction section, under the subsection ‘Storminess record’,

“The sedimentary record of core F06 shows repetitive fluctuations between periods of higher and lower input of aeolian sand to the site between 10,100 and 2,500 B.P. The bottom part of the sequence (unit I, ca. 10,100–6,800 B.P.), is first characterized by a marked succession of two wide peaks in both total and > 125 µm ASI separated by a clear low.”

now reads:

“The sedimentary record of core F06 shows repetitive fluctuations between periods of higher and lower input of aeolian sand to the site between 10,200 and 2,500 B.P. The bottom part of the sequence (unit I, ca. 10,200–6,800 B.P.), is first characterized by a marked succession of two wide peaks in both total and > 125 µm ASI separated by a clear low.”

and in the same section,

“Following the deposition of the sandy unit III (4,300–4,100 B.P.) and throughout most of unit IV, we observe a stable decrease of ASI, and notably of the ASI > 125 µm before it rises again sharply around 3,000 B.P.”

now reads:

“Following the deposition of the sandy unit III (4,300–4,100 B.P.) and throughout most of unit IV, we observe a stable decrease of ASI, and notably of the ASI > 125 µm before it rises again sharply around 3,200 B.P.”

Also in this section,

“The sedimentary record from Filsø can be divided into eight main periods of increased storminess (“Filsø Storm Periods” FSP) centred on 9,700–8,900 (FSP1), 8,700–8,400 (FSP2), 8,200–7,000 (FSP3), 6,600–6,700 (FSP4), 6,400–5,300 (FSP5), 5,100–4,500 (FSP6), 4,400–3,800 (FSP7) and 3,300–2,900 B.P. (FSP7) (Fig. 5A).”

now reads:

“The sedimentary record from Filsø can be divided into eight main periods of increased storminess (“Filsø Storm Periods” FSP) centred on 9,700–9,100 (FSP1), 8,800–8,600 (FSP2), 8,300–7,100 (FSP3), 6,900–6,700 (FSP4), 6,400–5,500 (FSP5), 5,100–4,700 (FSP6), 4,400–3,800 (FSP7) and 3,300–2,800 B.P. (FSP7) (Fig. 5A).”

Also in the Introduction, under the subsection ‘A multi-scale variability of Holocene North-Atlantic storminess activity’,

“Spectral analysis of the lower frequencies shows storminess to be composed of five periodicities of ≈ 2,500, ≈ 1,300, ≈ 600, ≈ 400 and ≈ 200 years at the 99% significance level (Fig. 3A-III), the ≈ 2,500-yr period showing the strongest power. As higher frequencies are concerned, strong wavelengths of ≈ 600-, ≈ 400-, ≈ 300-, ≈ 200-, ≈ 130- and ≈ 80-yr periods are evidenced (Fig. 3B-III), demonstrating the persistence of pluri-decadal to centennial scale modulations of North Atlantic storminess during the Holocene. EHA show variable stationarity of these millennial and centennial periodicities. A relatively wide band of quasi-periodic variability containing the dominant ≈ 2,500-yr period clearly dominates the signal over most of the Holocene (Fig. 3A-IV). A notable non-stationarity of the spectral signature nonetheless appears between ≈ 6,000 and ≈ 4,500 B.P., when the signal observes a marked excursion toward shorter periods of ≈ 1,300 years (Fig. 3A-IV). This temporary excursion towards a ≈ 1,300-yr mode of periodicity at the mid-Holocene seems accompanied by a convergence in the high-frequencies wavelengths characterizing the storminess signal during the early-Holocene (Fig. 3B-IV). Indeed, from ca. 10,000 to ca. 6,800–6,500 B.P, EHA show our storm record to be dominated by robust quasi-stationary ≈ 480-yr and ≈ 300-yr wavelengths (Fig. 3B-IV). A clear shift operates around 6,800–6,500 B.P. when the ≈ 280-yr periodicity is observed to progressively vanish while the ≈ 500-yr period evolves towards a strong ≈ 400-yr one. Pluri-millennial periodicities have been repeatedly documented in Holocene palaeo-environmental studies of the North Atlantic domain. The origins of these latter have been intensively discussed with regards to external (solar) and internal (oceanic/atmospheric) variability^30,31,32,33,34,35^. The ≈ 2,500-yr periodicity has been proposed to be strongly correlated with variations in the total solar irradiance^34,36^. The persistence of a clear and stationary ≈ 2,500-yr periodicity in our storm-record over most of the Holocene (Fig. 3A-IV) thus bring in contention that solar forcing may have been the main driver of the functioning of the climate system and storminess activity at a ca. 2,500-yr pace over the Holocene.”

now reads:

“Spectral analysis of the lower frequencies shows storminess to be composed of five periodicities of ≈ 2,200, ≈ 1,350, ≈ 590, ≈ 450 and ≈ 200 years at the 90% significance level (Fig. 3A-III), the ≈ 2,200-yr period showing the strongest power. As higher frequencies are concerned, strong wavelengths of ≈ 600-, ≈ 500-, ≈ 450-, ≈ 280-, ≈ 200-, ≈ 150- and ≈ 80-yr periods are evidenced (Fig. 3B-III), demonstrating the persistence of pluri-decadal to centennial scale modulations of North Atlantic storminess during the Holocene. EHA show variable stationarity of these millennial and centennial periodicities. A relatively wide band of quasi-periodic variability containing the dominant ≈ 2,200-yr period clearly dominates the signal over most of the Holocene (Fig. 3A-IV). A notable non-stationarity of the spectral signature nonetheless appears between ≈ 6,000 and ≈ 4,500 B.P., when the signal observes a marked excursion toward shorter periods of ≈ 1,300 years (Fig. 3A-IV). This temporary excursion towards a ≈ 1,300-yr mode of periodicity at the mid-Holocene seems accompanied by a divergence in the high-frequencies wavelengths characterizing the storminess signal during the early-Holocene (Fig. 3B-IV). Indeed, from ca. 10,200 to ca. 6,800 B.P, EHA show our storm record to be dominated by robust quasi-stationary ≈ 400 to 500-yr and 300-yr wavelength (Fig. 3B-IV). A clear shift operates around 6,800–6,500 B.P. when the ≈ 280-yr periodicity is observed to progressively vanish while the ≈ 500-yr period evolves towards strong ≈ 650-yr and 450 ones. Pluri-millennial periodicities have been repeatedly documented in Holocene palaeo-environmental studies of the North Atlantic domain. The origins of these latter have been intensively discussed with regards to external (solar) and internal (oceanic/atmospheric) variability.^30,31,32,33,34,35^ Periodicities of ca. 2000–2,500 years have been proposed to be strongly correlated with variations in the total solar irradiance^34,36^. The persistence of a clear and stationary ≈ 2,200-yr periodicity in our storm-record over most of the Holocene (Fig. 3A-IV) thus bring in contention that solar forcing may have been the main driver of the functioning of the climate system and storminess activity at a ca. 2,500-yr pace over the Holocene.”

The original version of Figure 3 was incorrect and appears below as Figure 2. Figure 3 has been corrected in the original version of the Article where Curves A-I, A-II, B-I and B-II have been replaced by the ASI curves corrected from the mistake in the age-depth model. Plots A-III, A-IV, B-III and B-IV have been changed accordingly to the results of the MTM and EHA analyses performed on the corrected ASI time-series. New diagrams showing the AR-1 confidence levels and the MTM F-test have also been added to both A and B.

**Figure 2:**
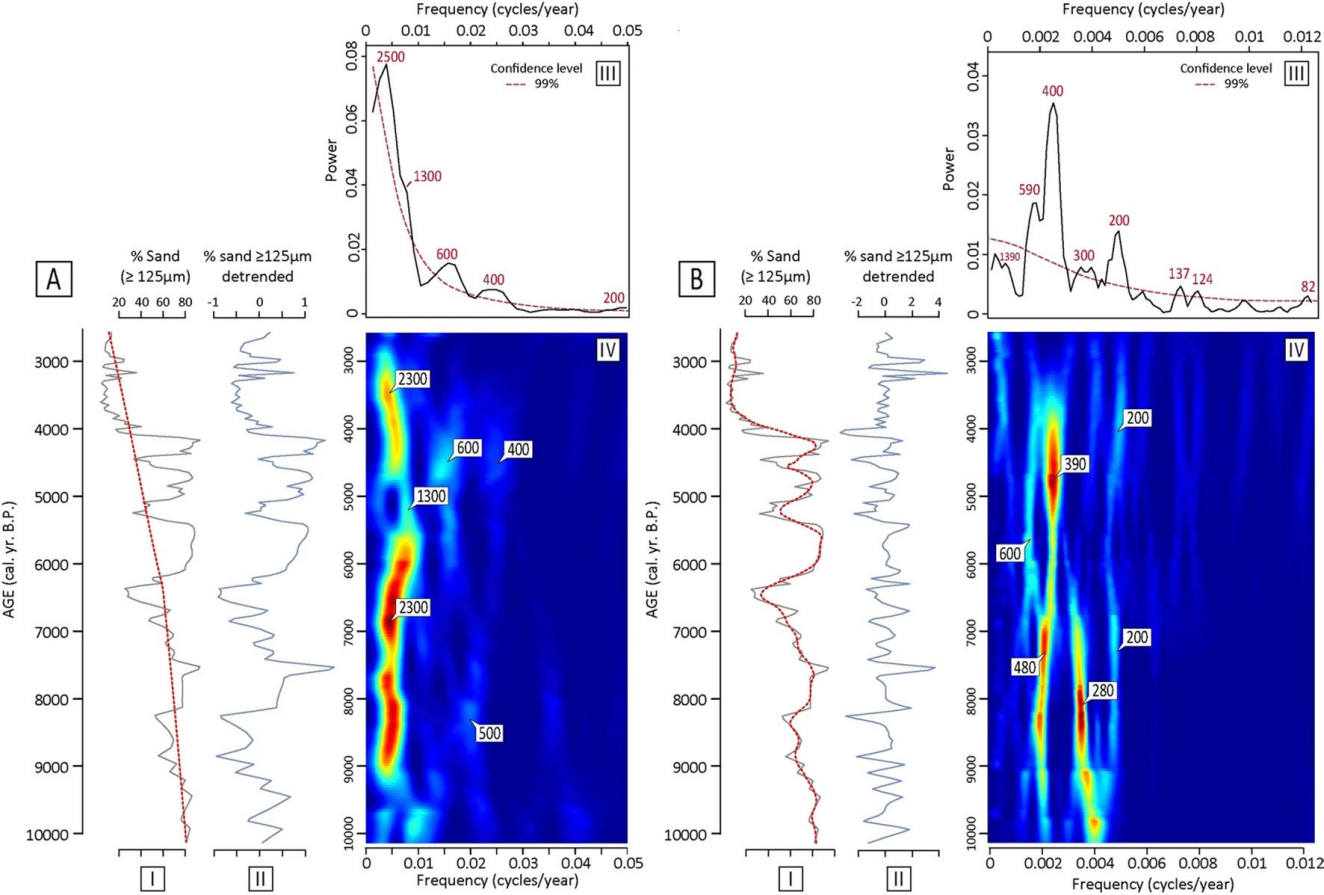
.

In the Introduction section, under the subheading ‘North-Atlantic storminess and ocean dynamics linkages.’,

“Multi-taper coherence analyses (CMTM, see methods) performed on our storm record and on the record of the abundance of G.ruber w. show the two series to share common periodicities of ≈ 1,500, ≈ 1,000, ≈ 750, ≈ 400, ≈ 300 and ≈ 200 yrs, the highest coherence estimates being obtained for the ≈ 300 and ≈ 200-yr periods (Fig. 5B).”

now reads:

“Multi-taper coherence analyses (CMTM, see methods) performed on our storm record and on the record of the abundance of G.ruber w. show the two series to share common periodicities of ≈ 1,300, ≈ 700, ≈ 400, ≈ 240 yrs, the highest coherence estimates being obtained for the ≈ 1,300, 700 and ≈ 400-yr periods (Fig. 5B).”

Also in this section,

“The results of the coherence analyses between storminess and the proxy record of the extent of the STG53 nonetheless show clear positive phasings (of 67-, 7-, 69- and 35-degrees, respectively) at centennial time-scales (Fig. 5B). Such phasing suggests that changes in storminess led STG variability at centennial time-scales, in line with what is observed nowadays at inter-annual to decadal scales^4^. The opposite appears in longer periods, the slight anti-phasing observed at the ≈ 1,500, ≈ 1,000 and ≈ 760-yr period (− 27 degrees, Fig. 5B) being in favour of a bottom-up forcing of oceanic variability on atmospheric processes at millennial time-scales.”

now reads:

“The results of the coherence analyses between storminess and the proxy record of the extent of the STG53 nonetheless show clear positive phasing (of 13, 95 and 28-degrees, respectively) at both millennial and centennial time-scales (Fig. 5B). Such phasing suggests that changes in storminess led STG variability, in line with what is observed nowadays at inter-annual to decadal scales^4^. Smaller phases are obtained for ≈ 1,300 and 700-yr periods (13 and 0 degrees, respectively, Fig. 5B) being in favour of a bottom-up forcing of oceanic variability on atmospheric processes at millennial time-scales.”

Also in this section,

“A nearly perfect peak to peak synchronism is also apparent between our record and the stacked record of Ice-Rafted Debris (IRD) reconstructed in the North Atlantic over the 10,000–2,500 B.P. period^31^ (“Bond events” 6, 5, 4 and 3, Noted “BE” on Fig. 5A-II).”

now reads:

“A nearly perfect peak to peak synchronism is also apparent between our record and the stacked record of Ice-Rafted Debris (IRD) reconstructed in the North Atlantic over the 10,200–2,500 B.P. period^31^ (“Bond events” 6, 5, 4 and 3, Noted “BE” on Fig. 5A-II).”

Also in this section,

“Multi-taper coherence analyses of our storm record and the input of IRD to the North-eastern Atlantic show the two series to be highly coherent at the 1,500-yr period with a phase lag of 35 degrees (Fig. 5C).

now reads:

“Multi-taper coherence analyses of our storm record and the input of IRD to the North-eastern Atlantic show the two series to be highly coherent at the 1,500-yr period with a phase lag of 47 degrees (Fig. 5C).

The original version of Figure 5 was incorrect and appears below as Figure 3. Figure 5 has been corrected in the original version of the Article. The curve on Fig. 5 A-1 was changed accordingly to the correction of the ASI time-series. Some modifications were made to the vertical grey band FSP-7 defining the “Filso storm period number 7”, in order to adapt to the modifications brought by the correction to the ASI curve between ca. 4,000 and 2,500 cal. B.P. In Fig. 5-B and 5-C: ASI curves (red curves) were replaced on each of the plot by the new ASI curves corrected from the age-model mistake. Blue and green curves were changed to match the new results of the coherence analyses performed on the ASI time-series corrected form the age model mistake. Numbers indicating the statistically significant coherent periodicities and their respective phases were modified accordingly to the results of the new coherence analyses.

**Figure 3:**
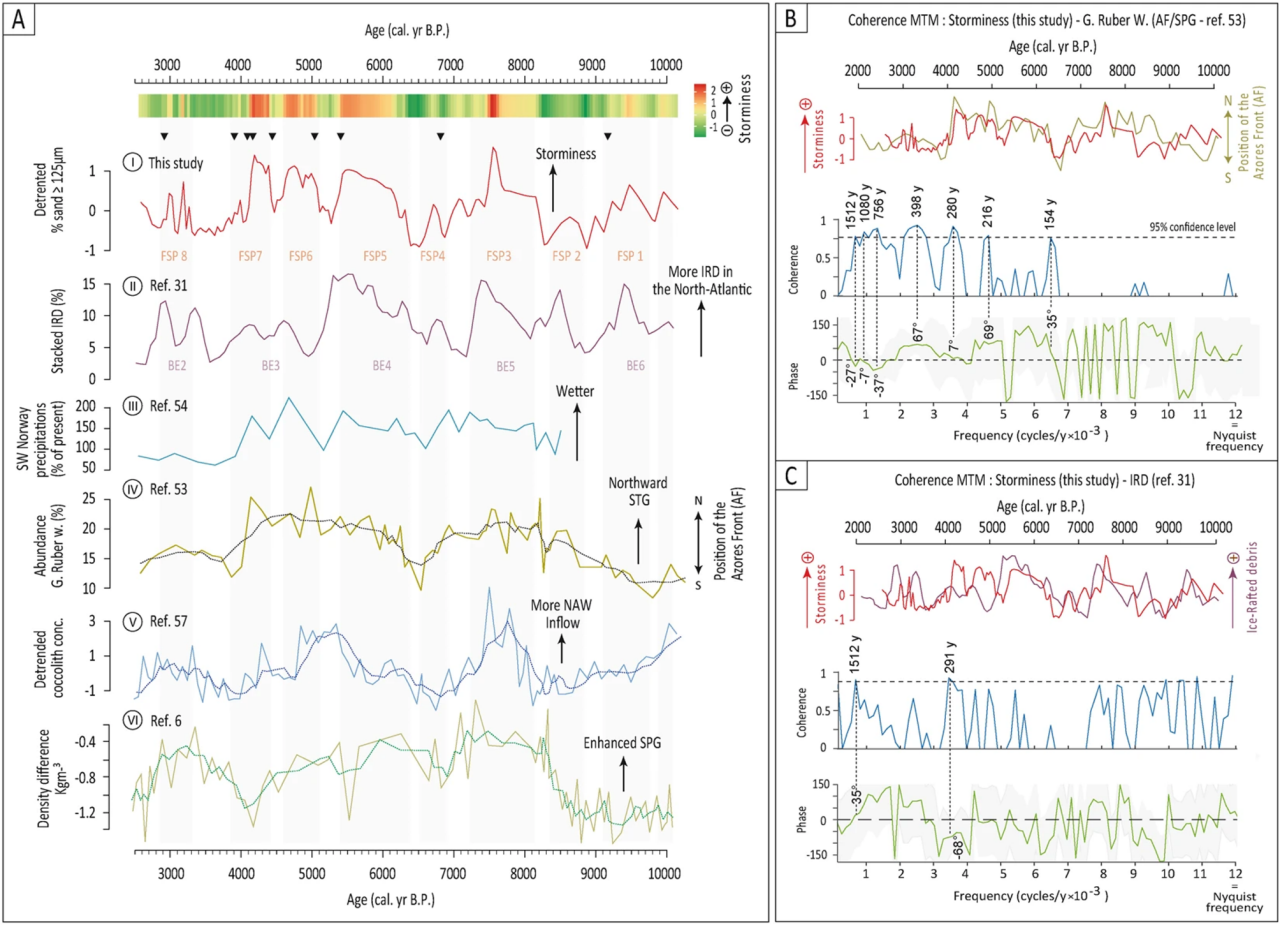
.

In the Conclusion,

“Storminess most likely drove the activity of North-Atlantic oceanic gyres at centennial time-scales but the ocean appears to have influenced back atmospheric variability at longer millennial time-scales.”

now reads:

“Storminess most likely drove the activity of North-Atlantic oceanic gyres at both millennial and centennial time-scales.”

In the Methods section, under the subheading ‘Statistical analyses’,

“All time-series were linearly re-interpolated at 40 yrs from 2,580 to 10,100 yrs B.P. prior to the CMTM.”

now reads:

“All time-series were linearly re-interpolated at 40 yrs from 2,488 to 10,200 yrs B.P. prior to the CMTM.”

The original version of the Supplementary Information file also contained errors.

In Supp. Info 2 changes have been made to Core F06 (left-hand side) and Core F02 (right-hand side). In Core F06 (left-hand side), the date noted for F06-U on the original figure was renamed F06-6 and annotated "rejected". Date F06-4 was recalibrated. Its position alongcore, originally mistakenly marked at 176 was changed to 181 and annotated “rejected”. In Core F02 (right-hand side) the date originally noted F02-6 was modified to read 2,783 (2,739) 2,505 and renamed F02-4. The date originally noted F02-5 was renamed F02-3 and annotated “rejected”. All dates were re-numbered from F02-1, instead of being numbered from F02-3 in the original figure.

Supp. Info 3A has been modified where the topmost date has been changed. The date at 181 cm depth was changed to match the re-calibration of date F06-4. The curve of the age-model was redrawn to match the new age-model.

Supp. Info 3B has been modified so that the dates from the two cores are mixed and presented in chronological order; as they appear in the compound stratigraphic model: the younger age on top, the older at the bottom. Three new columns were added; (i) a column showing the depth of the datation in the core from which it was obtained; (ii) an accepted/rejected column so that rejected datations are more easy to find by the reader and (iii) a column showing the position of the datation in the compound age-depth model. In the rightmost column ‘Age [cal yr BP]’, the authors added the median age (in brackets) to the minimum and maximum ages given by the two-sigma probabilities. Some ages were not reported with their full error ranges in the original version of this table so all ages were recalibrated following a unique procedure under calib 7.0 software and reported with the full 95.4% 2-sigma error range to ensure coherence and repeatability. This lead to slight changes in the min–max ages, but not in the median age.

These errors have now been corrected in the PDF and HTML versions of the Article, and in the accompanying Supplementary Information file. The original Supplementary Information accompanies this correction.

The overall conclusions of the Article are unaffected by these corrections.

## Supplementary information

Supplementary Information.

