# Supplementary material for: Author Correction: Holocene centennial to millennial shifts in North-Atlantic storminess and ocean dynamics
Source: Sci Rep. 2020 Aug 6;10:13529. doi: 10.1038/s41598-020-69870-7 (PMC7414029; doi:10.1038/s41598-020-69870-7)
Supplement: Supplementary file 1 — Supplementary Information. [file 41598_2020_69870_MOESM1_ESM.docx]

**SUPPLEMENTARY ONLINE INFORMATION**

**Holocene centennial to millennial shifts in North-Atlantic storminess and ocean dynamics**

Jérôme Goslin^1*^, Mikkel Fruergaard^1^, Lasse Sander^2^, Mariusz Gałka^3^,

Laurie Menviel^4^, Johannes Monkenbusch^1^, Nicolas Thibault^1^ & Lars B. Clemmensen^1^


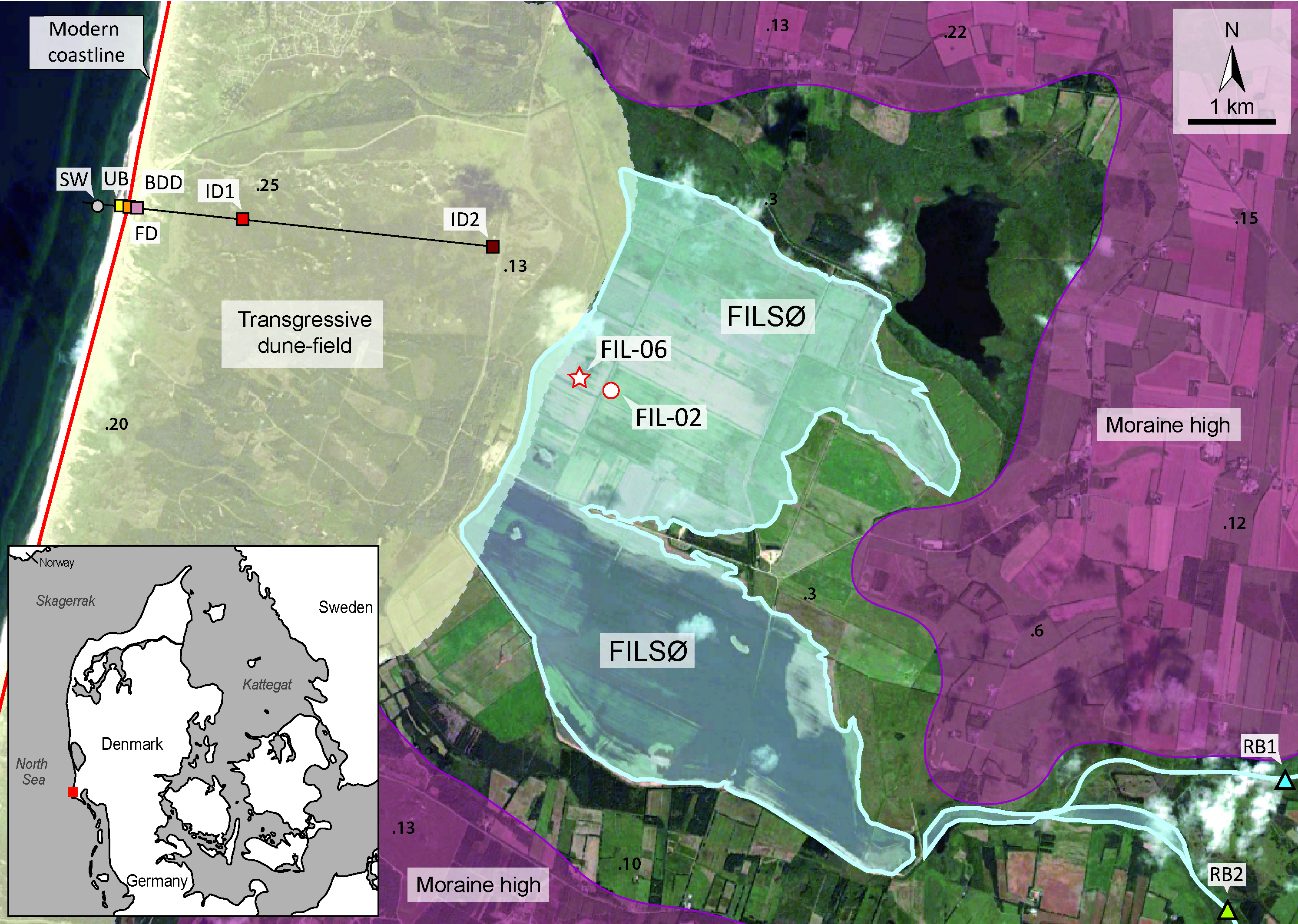


**Supp. Info 1.** Location map of Filsø area (western Denmark). Dotted number spread on the map indicates elevation (in meters DVR90). Red circled signs show the positions of the cores used in this study. The red star indicates the location of core F-06 from which the past-storminess reconstruction presented in the present study was derived. The red circle shows the location of core F-02 which was used as a supplementary core during the construction of the age model (see supp. Info. 2 and 3). Are also shown the location of the modern beach/dunes and river surface sand samples used for the geochemical (µ-XRF) characterization of the potential sources of the sand observed within core F-06 (see supp. Inf. 5). SW=beach face / swash zone, UB= Upper Beach, FD=Frontal Dune, BDD= Back Dune Deposits, ID=Internal Dunes, RB=River Bed. Figure produced using Adobe illustrator CS3. The satellite image is reproduced with the permission of the data provider (Google Earth, DigitalGlobe 2018) and is published under a CC BY license granted to Macmillan Publishers Ltd, part of Springer Nature. Note that the image was taken while Filsø was still reclaimed for agricultural purposes. The blue shape indicates the area of the lake at the time of core collection.

**SUPPLEMENTARY ONLINE INFORMATION**

**Holocene centennial to millennial shifts in North-Atlantic storminess and ocean dynamics**

Jérôme Goslin^1*^, Mikkel Fruergaard^1^, Lasse Sander^2^, Mariusz Gałka^3^,

Laurie Menviel^4^, Johannes Monkenbusch^1^, Nicolas Thibault^1^ & Lars B. Clemmensen^1^

**
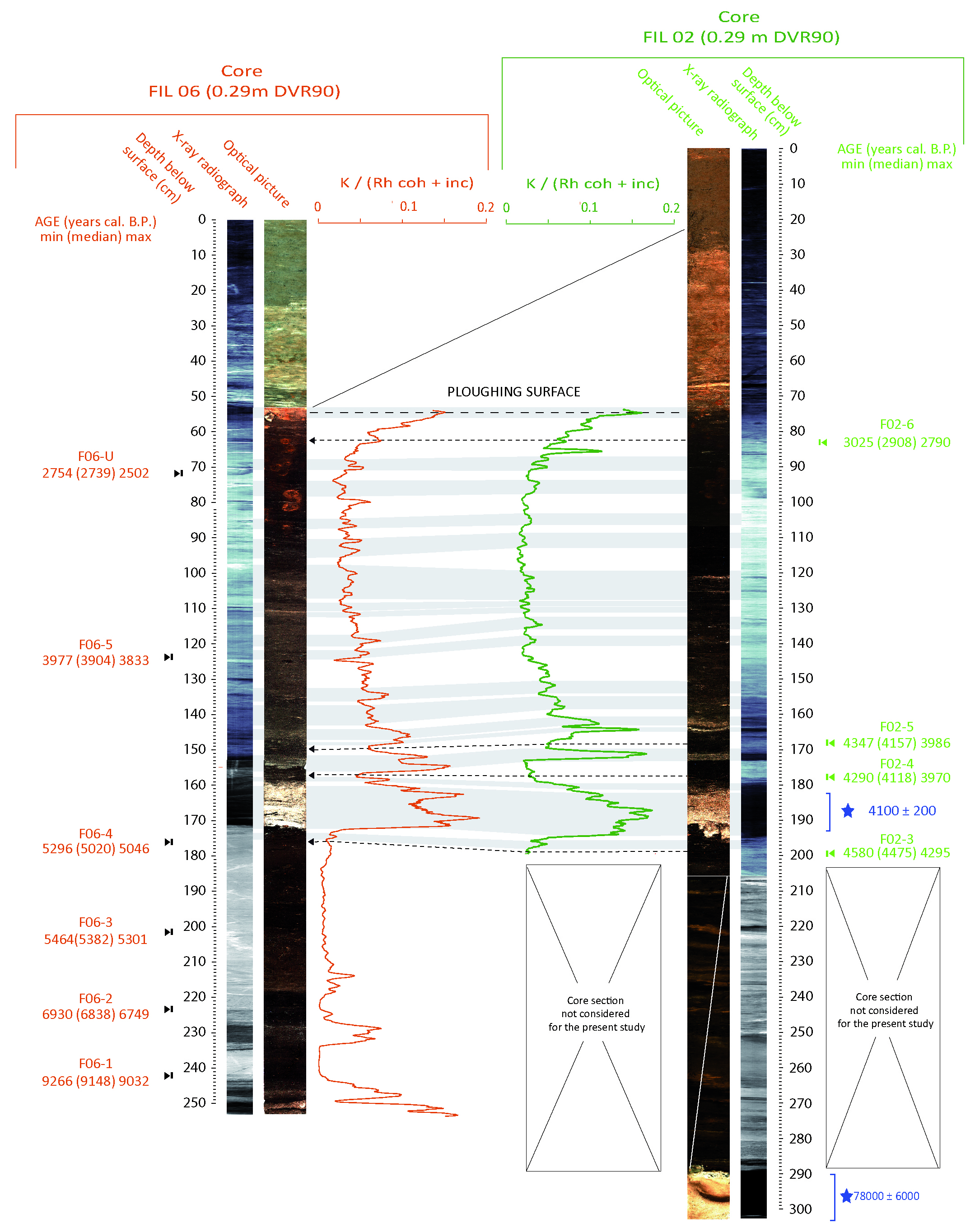
**

**Supp. Info 2.** Optical pictures and X-Ray radiograph of cores F-06 (left) and F-02 (right), showing the stratigraphy of the sequence retrieved by the cores. µ-XRF potassium (K) downcore profiles are shown for each core. These were used for linking both cores in order to use supplementary dates from core F-02 in the building of core F-06 age-model (see Supp. Info. 3). Note the close correspondence of the signal between the two cores. Position of AMS radiocarbon age controls (orange and green ages, in years cal. B.P.) and OSL ages (blue, in years B.P.) are indicated.

**SUPPLEMENTARY ONLINE INFORMATION**

**Holocene centennial to millennial shifts in North-Atlantic storminess and ocean dynamics**

Jérôme Goslin^1*^, Mikkel Fruergaard^1^, Lasse Sander^2^, Mariusz Gałka^3^,

Laurie Menviel^4^, Johannes Monkenbusch^1^, Nicolas Thibault^1^ & Lars B. Clemmensen^1^

**
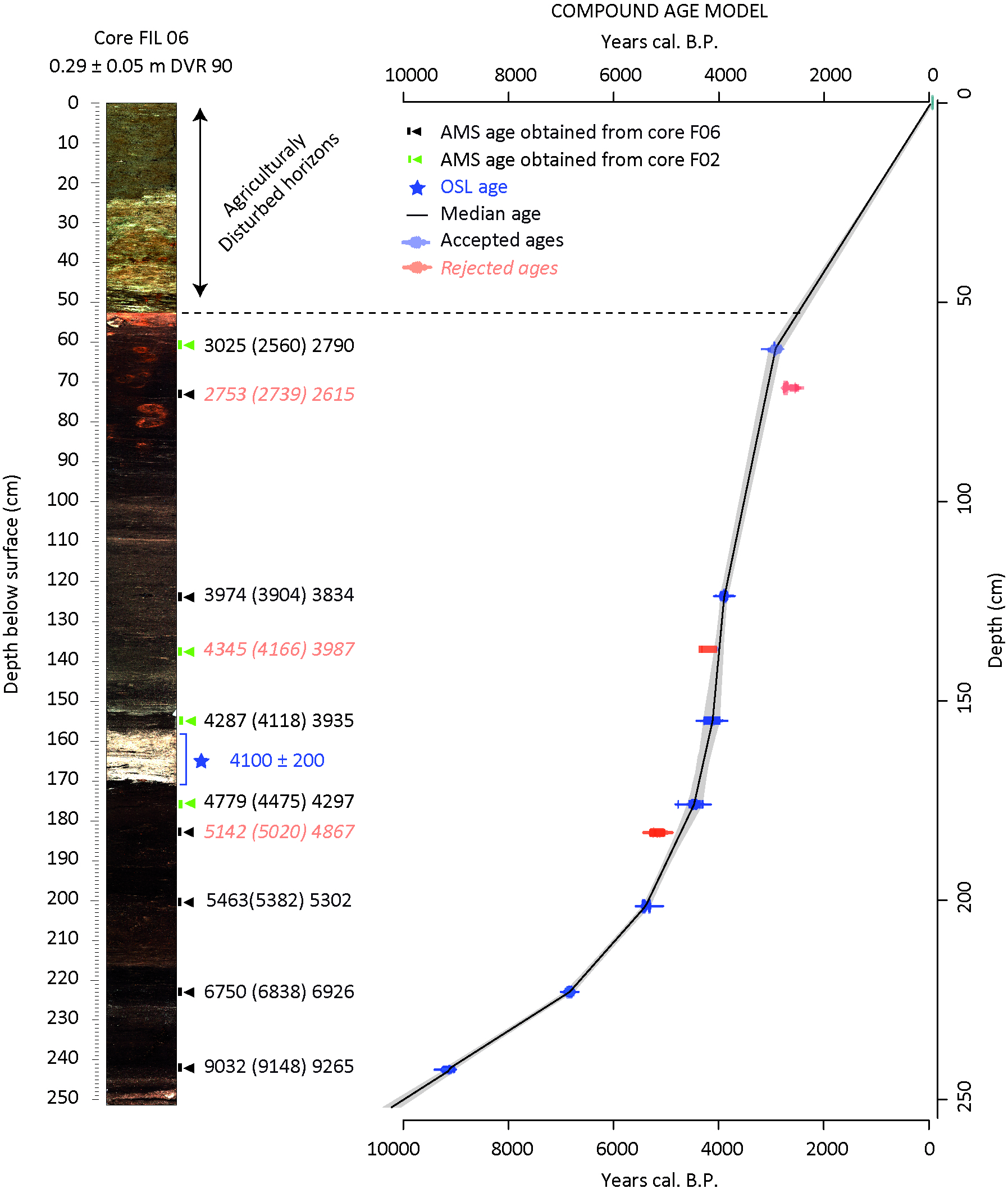
**

**Supp. Info 3A.** Age-model used in this study to reconstruct the chronology of Holocene storminess over western Denmark.

| Sample | Material | Lab. code - number | C14 date [BP] | Age [cal yr BP] (95.4%) |
| --- | --- | --- | --- | --- |
| F06-U | Unidentified plant fragments | Beta480168 | 2560 ± 30 | 2754-2502 |
| F06-5 | *Carex* sp. fruits x2, cf. *Schoenoplectus* sp. fruit, *Betula* sect. Alba fruit x3, bud scale, wood pieces | Beta480167 | 3590 ± 30 | 3977-3833 |
| F06-4 | *Schoenoplectus* *tabernaemontani* sp. fruit, *Mentha aquatic*a seeds x4, *Carex* sp. fruits x6, *Polygonum* sp. fruits x2, *Hippuris vulgaris* seeds x2, *Cicuta virosa* fruit Wood pieces, *Carex* sp. fruit, stem bases | Beta480166 | 4500 ± 30 | 5296-5046 |
| F06-3 | *Hydrocotyle vulgaris* seed x1, charcoal pieces, stem bases | Beta480165 | 4630 ± 30 | 5464-5301 |
| F06-2 | Charcoal pieces | Beta480164 | 6000 ± 30 | 6930-6749 |
| F06-1 | Charcoal pieces | Beta480163 | 8200 ± 30 | 9266-9032 |
| F02-5 | *Betula sp. fruits*, *Sphagnum sp.* stems | LuS12578 | 3780 ± 45 | 4300-3985 |
| F02-4 | *Betula sp. fruits* and leaves | LuS12249 | 3755 ± 50 | 4290-3970 |
| F02-3 | *Betula sp. fruits*, *Sphagnum sp.* leaves and stems | LuS12247 | 3995 ± 45 | 4580-4295 |

| Sample | Depth | Risø lab. number | Age , ka | Dose , Gy | (n) | Dose rate, Gy/ka | w.c. % |
| --- | --- | --- | --- | --- | --- | --- | --- |
| Filsø OSL 1 | F02 183-190 | 17 47 01 | 4.1 ± 0.2 | 3.24 ± 0.11 | 22 | 0.78 ± 0.03 | 25 |
| Filsø OSL 2 | F02 290-300 | 17 47 02 | 78 ± 6 | 65 ± 3 | 19 | 0.84 ± 0.04 | 25 |

**Supp. Info 3B.** Tables of the radiocarbon dates (top) and OSL ages (bottom) produced and used within this study.

**SUPPLEMENTARY ONLINE INFORMATION**

**Holocene centennial to millennial shifts in North-Atlantic storminess and ocean dynamics**

Jérôme Goslin^1*^, Mikkel Fruergaard^1^, Lasse Sander^2^, Mariusz Gałka^3^,

Laurie Menviel^4^, Johannes Monkenbusch^1^, Nicolas Thibault^1^ & Lars B. Clemmensen^1^

**
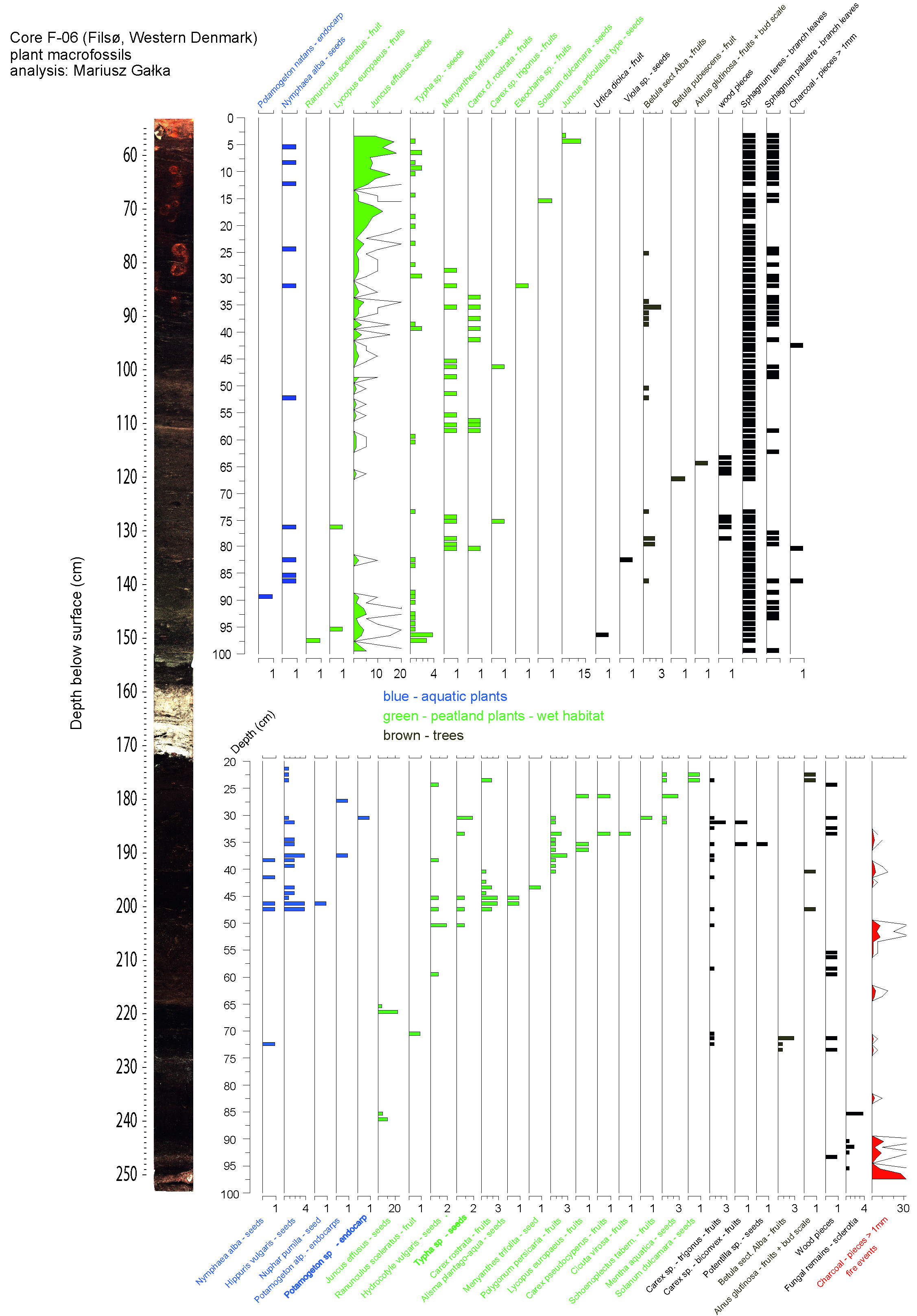
**

**Supp. Info 4.** Plant macrofossils diagrams of core F-06.

**SUPPLEMENTARY ONLINE INFORMATION**

**Holocene centennial to millennial shifts in North-Atlantic storminess and ocean dynamics**

Jérôme Goslin^1*^, Mikkel Fruergaard^1^, Lasse Sander^2^, Mariusz Gałka^3^,

Laurie Menviel^4^, Johannes Monkenbusch^1^, Nicolas Thibault^1^ & Lars B. Clemmensen^1^


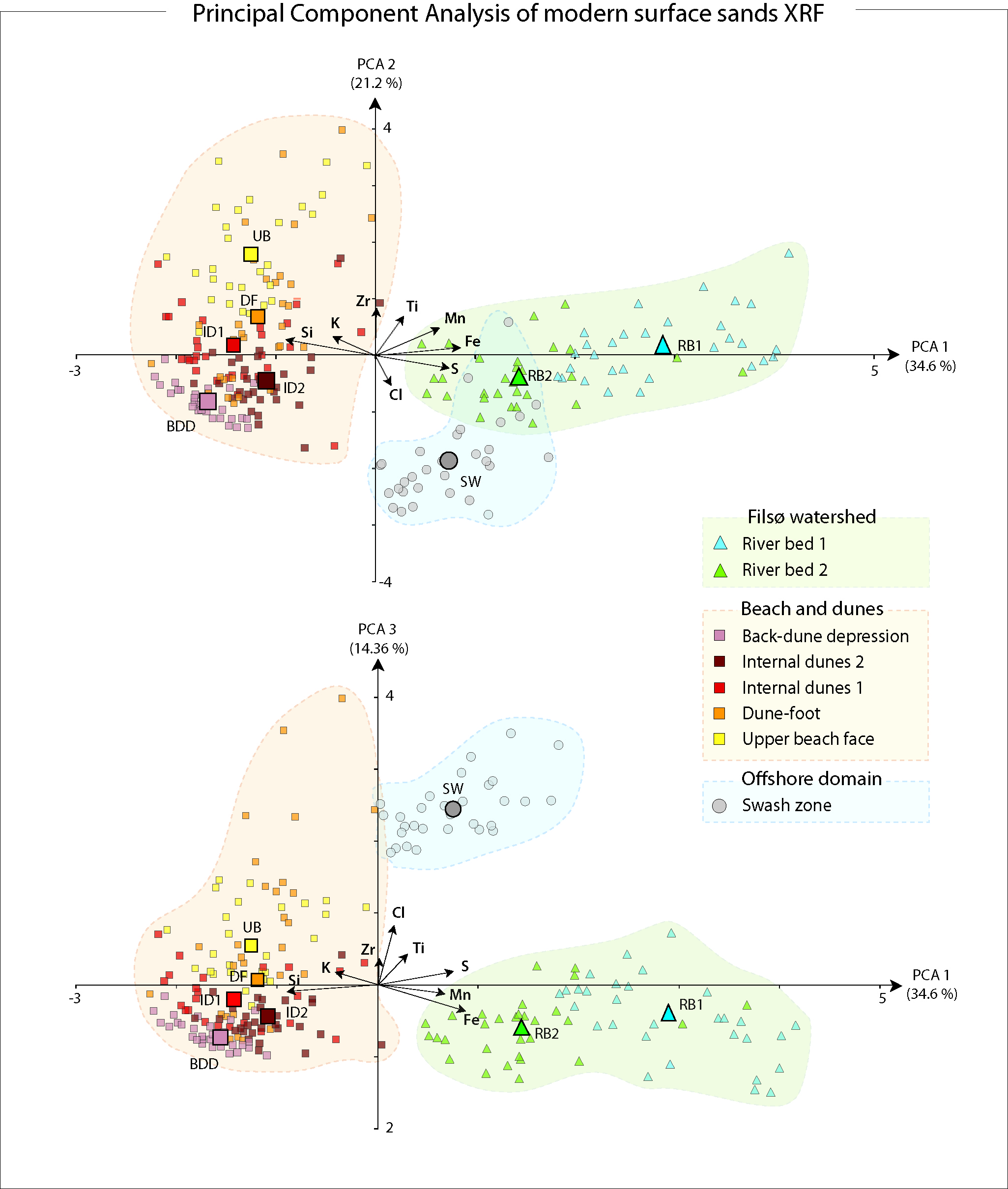


**Supp. Info 5.** Results of the Principal Component Analysis (PCA) of modern Beach, dunes and rivers surface sand samples retrieved from our study area (see the sampling locations in Supp. Info. 1). Small colored symbols indicate the position of each observation (1 point= 1 µ-XRF analysis).Larger symbols show the mean contribution of each deposit environment to the PCA-score. XRF data were normalized by Rh inc+coh to account for variations in water content and organic content between the samples ^63^. Results were then were centered and reduced prior to the PCA by substracting to each result the mean of the results given for each element and by dividing by the standard deviation. This differentiation between beach/dune sand and river sand may be explained by a higher presence of mafic silicates of K-Feldspar with beach and dunes sands, while higher Fe contents in river sands may originates from higher concentration of magnetite-sourced material (originating from local Till deposits) and/or pyrite coatings. The exploration of PCA-3 demonstrates the proximity of the swash-zone sand to other beach and dune samples, the difference being mostly driven by higher chlorine content.

**SUPPLEMENTARY ONLINE INFORMATION**

**Holocene centennial to millennial shifts in North-Atlantic storminess and ocean dynamics**

Jérôme Goslin^1*^, Mikkel Fruergaard^1^, Lasse Sander^2^, Mariusz Gałka^3^,

Laurie Menviel^4^, Johannes Monkenbusch^1^, Nicolas Thibault^1^ & Lars B. Clemmensen^1^

**
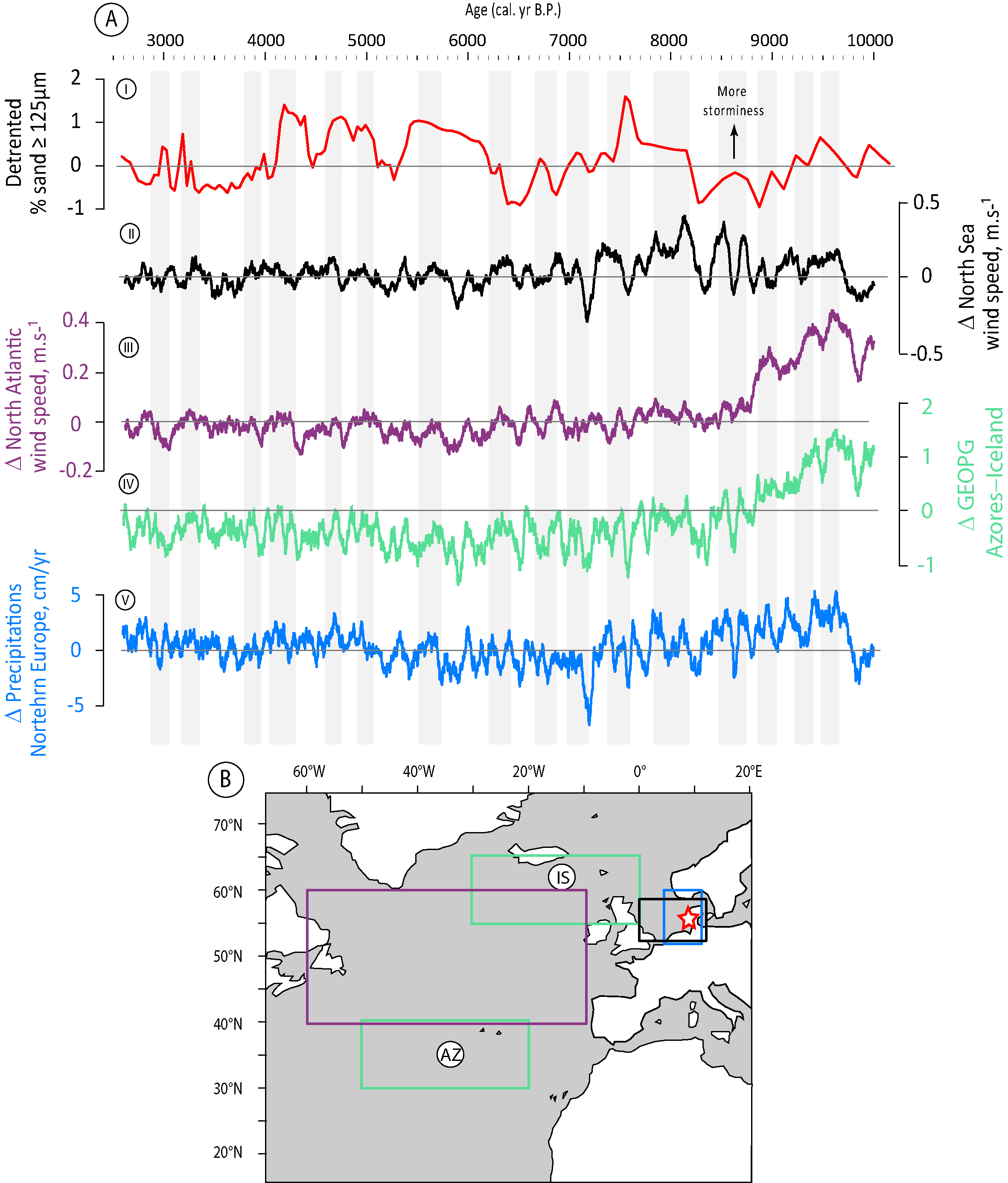
**

**Supp. Info 6.** **(A-I)** Storminess record from this study and **(AII to V)** Time series produced by transient simulations of the Holocene with the Earth system model LOVECLIM (Kobashi *et al.*, 2017). 100-years moving averages. **(A-II)** Timeseries of wind speed anomalies averaged over the North Sea (0-12E, 54-58N, m/s, Black box on B), **(A-III)** Timeseries of wind speed anomalies averaged over the North Atlantic (60W-10W,40-60N, m/s, Red box on B), **(A-IV)** Timeseries of 500hPa geopotential height difference between the Azores and Iceland (Green boxes on B), (A-V) Timeseries of precipitation anomalies averaged over northern Europe (6:11E, 53:60N, cm/yr, Blue box on B).
